# miR‐195 reduces age‐related blood–brain barrier leakage caused by thrombospondin‐1‐mediated selective autophagy

**DOI:** 10.1111/acel.13236

**Published:** 2020-10-07

**Authors:** Chien‐Yuan Chen, Yung‐Mei Chao, Hsiu‐Fen Lin, Chao‐Jung Chen, Cheng‐Sheng Chen, Jenq‐Lin Yang, Julie Y. H. Chan, Suh‐Hang H. Juo

**Affiliations:** ^1^ Graduate Institute of Medicine College of Medicine, Kaohsiung Medical University Kaohsiung Taiwan; ^2^ Institute for Translational Research in Biomedicine Chang Gung Memorial Hospital Kaohsiung Taiwan; ^3^ Department of Neurology Kaohsiung Medical University Hospital Kaohsiung Taiwan; ^4^ Department of Neurology College of Medicine, Kaohsiung Medical University Kaohsiung Taiwan; ^5^ Proteomics Core Laboratory Department of Medical Research, China Medical University Hospital Taichung Taiwan; ^6^ Graduate Institute of Integrated Medicine China Medical University Taichung Taiwan; ^7^ Department of Psychiatry Kaohsiung Medical University Hospital Kaohsiung Taiwan; ^8^ Department of Psychiatry College of Medicine, Kaohsiung Medical University Kaohsiung Taiwan; ^9^ Department of Medical Research China Medical University Hospital Taichung Taiwan; ^10^ Graduate Institute of Biomedical Sciences China Medical University Taichung Taiwan; ^11^ Institute of New Drug Development China Medical University Taichung Taiwan; ^12^ Drug Development Center China Medical University Taichung Taiwan

**Keywords:** blood–brain barrier, miR‐195, selective autophagy, thrombospondin‐1, tight junction

## Abstract

Blood–brain barrier (BBB) disruption contributes to neurodegenerative diseases. Loss of tight junction (TJ) proteins in cerebral endothelial cells (ECs) is a leading cause of BBB breakdown. We recently reported that miR‐195 provides vasoprotection, which urges us to explore the role of miR‐195 in BBB integrity. Here, we found cerebral miR‐195 levels decreased with age, and BBB leakage was significantly increased in miR‐195 knockout mice. Furthermore, exosomes from miR‐195‐enriched astrocytes increased endothelial TJ proteins and improved BBB integrity. To decipher how miR‐195 promoted BBB integrity, we first demonstrated that TJ proteins were metabolized via autophagic–lysosomal pathway and the autophagic adaptor p62 was necessary to promote TJ protein degradation in cerebral ECs. Next, proteomic analysis of exosomes revealed miR‐195‐suppressed thrombospondin‐1 (TSP1) as a major contributor to BBB disruption. Moreover, TSP1 was demonstrated to activate selective autophagy of TJ proteins by increasing the formation of claudin‐5‐p62 and ZO1‐p62 complexes in cerebral ECs while TSP1 impaired general autophagy. Delivering TSP1 antibody into the circulation showed dose‐dependent reduction of BBB leakage by 20%–40% in 25‐month‐old mice. Intravenous or intracerebroventricular injection of miR‐195 rescued TSP1‐induced BBB leakage. Dementia patients with BBB damage had higher levels of serum TSP1 compared to those without BBB damage (*p* = 0.0015), while the normal subjects had the lowest TSP1 (*p* < 0.0001). Taken together, the study implies that TSP1‐regulated selective autophagy facilitates the degradation of TJ proteins and weakens BBB integrity. An adequate level of miR‐195 can suppress the autophagy–lysosome pathway via a reduction of TSP1, which may be important for maintaining BBB function.

## Graphical abstract

The present study revealed that thrombospondin‐1 (TSP1) is a key factor for BBB leakage and miR‐195 protects BBB integrity. We showed that cerebral miR‐195 reduces with age and blood‐brain barrier (BBB) leakage is increased in miR‐195 knockout mice. miR‐195‐regulated thrombospondin‐1 impairs BBB via selective autophagy of tight junctions. Furthermore, circulating TSP1 was higher in BBB‐damaged patients, which implied TSP1 as a new biomarker of BBB damage.

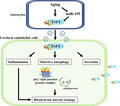

## 1 INTRODUCTION

Blood–brain barrier (BBB) leakage contributes significantly to the development of neurodegenerative diseases during aging process (Iadecola, 2015; Oakley & Tharakan, 2014). The BBB is formed by microvascular endothelial cells (ECs), surrounded by the basal lamina, pericytes, and astrocytic perivascular end‐feet. Endothelial tight junction (TJ) proteins seal brain endothelia to form the anatomical barrier. The deficiency of TJ proteins is associated with BBB breakdown in many neurological disorders (Zlokovic, 2011). Among TJ proteins, claudin‐5 and zonula occludens‐1 (ZO‐1) are mostly investigated for BBB dysfunction that is accompanied by the cell–cell communication disorder (Shindo et al., 2016; Zlokovic, 2011).

Our group has previously reported that miR‐195 plays an important role in vascular functions by improving endothelial function and suppressing inflammation (Cheng et al., 2019; Wang et al., 2012). Furthermore, our recent study showed that cerebral miR‐195 levels were decreased in stroke and supplement of exogenous miR‐195 improved stroke recovery and reduced brain damage (Cheng et al., 2019). Therefore, it is reasonable to postulate that miR‐195 may play a role in regulating BBB functions.

Thrombospondin‐1 (TSP1) is a large multidomain glycoprotein that participates in cell–cell interactions and can be secreted by multiple cells including astrocytes and ECs (Lu & Kipnis, 2010). TSP1 has been shown to contribute to the development of inflammation that led to EC dysfunction by interacting with its receptor CD36 (Liu et al., 2015). Recent studies showed that CD36 is also involved in cellular autophagy (Sanjurjo et al., 2015), suggesting the potential of TSP1–CD36–autophagy pathway in the metabolism of TJ proteins.

General autophagy is a nonselective degradation pathway for cellular homeostasis. However, accumulating evidence has highlighted that selective autophagy accounts for cellular metabolic disorders and neurodegenerative diseases (Stolz, Ernst, & Dikic, 2014). Selective autophagy is a specific degradation of proteins and organelles via autophagic–lysosomal pathway. Specificity for selective degradation depends on the direct binding of target cargo (e.g., TJ proteins) and autophagic adaptors (e.g., p62) (Mancias & Kimmelman, 2016). The autophagic adaptor–cargo complex also binds to microtubule‐associated light chain 3 (LC3) to facilitate the uptake of this complex into autophagosome. Recent studies suggested that selective autophagy can be activated despite dysfunctional autophagy in the brain (Lim et al., 2015), which implies selective autophagy may play a role in BBB dysfunction.

Astrocytes provide the required support for BBB maintenance. In astrocyte–EC cross talk, astrocytes release exosomes as one of the mechanisms for intercellular communication (Guescini, Genedani, Stocchi, & Agnati, 2010). Exosome components including miRNA, mRNA, and protein can modulate the properties of brain ECs. Previous studies indicated that astrocyte‐derived exosomes suppressed vessel leakage in the retina (Hajrasouliha et al., 2013). By investigating the astrocyte exosomal components, we identified miR‐195‐regulated TSP1 as a major contributor to BBB leakage. The detailed mechanisms and pivotal role of TSP1 in BBB leakage were illustrated in cells, animals, and human samples in the present study.

## 2 RESULTS

### 2.1 Low miR‐195 level leads to BBB leakage

**Figure 1:**
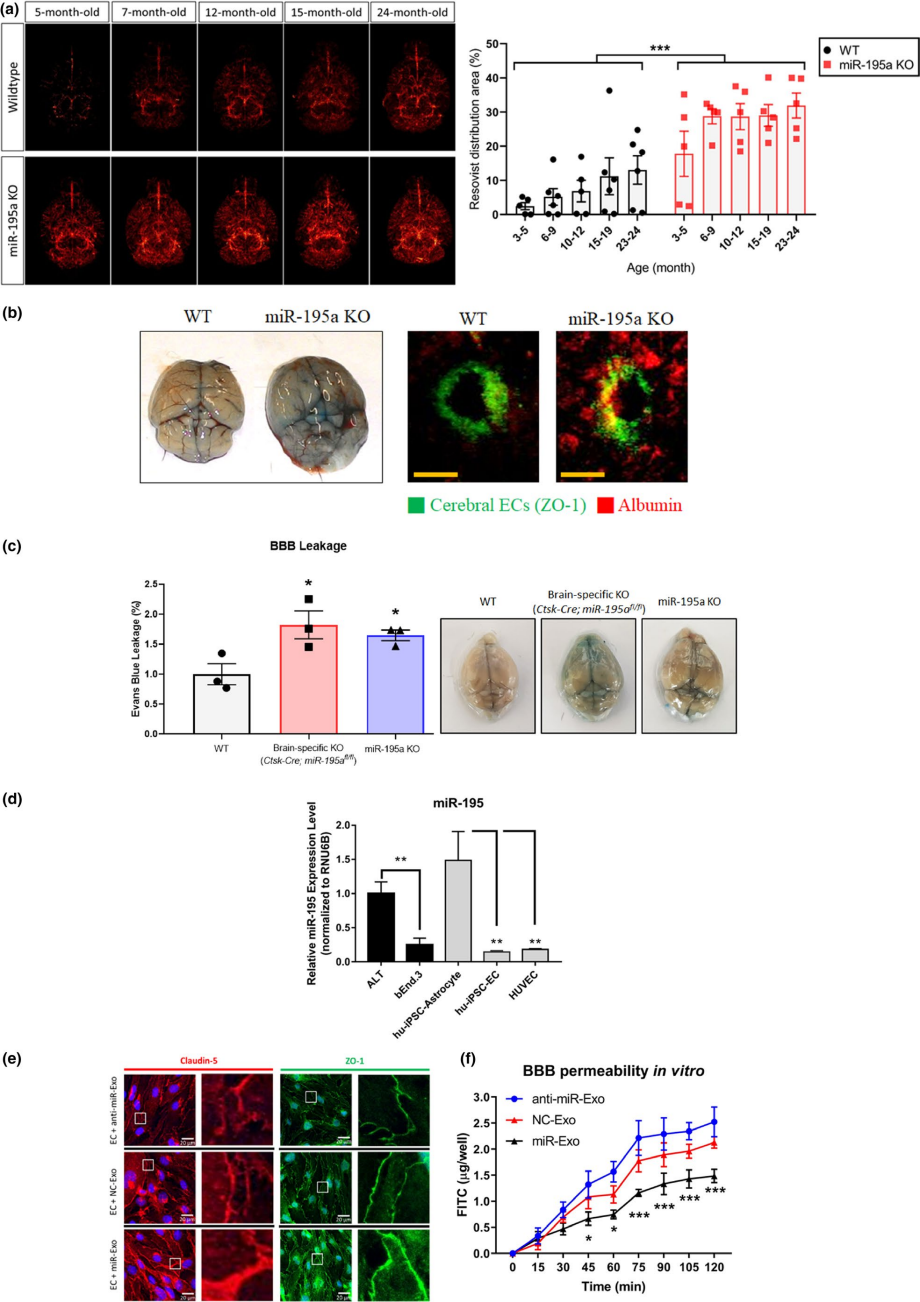
miR‐195 enhanced BBB integrity via astrocyte‐derived‐exosomes. (a) MRI images showing more BBB leakage of miR‐195a knockout (KO) mice compared to wild‐type (WT) mice regardless of age. The intensity of the red signals represents the amount of Resovist extravasation in the brain parenchyma which implicated the severity of BBB leakage. ***p < 0.001, *n* = 5–6/group. (b) Representative images showing the Evans blue extravasation in the whole brain (left panel) and serum albumin extravasation (red) in the brain parenchyma (right panel) of miR‐195a KO mice and the WT littermate at the age of 4 months. Cerebral ECs were stained for ZO‐1 (green). Magnification: 200×. Scale bar = 20 μm. See quantitative data in Figure S1C. (*n* = 3/group) (c) BBB permeability measured by Evans blue assay in WT, brain‐specific KO (*Ctsk*‐*Cre*;*miR*‐*195a^fl^* ^/^ *^fl^*), and miR‐195a KO mice aged 12 months (left panel). Data are presented as mean ± *SEM*, *p < 0.05, *n* = 3/group. Representative images show Evans blue extravasation in whole brains of mice of three types (right panel). (d) miR‐195 levels in astrocytes and ECs. Data are presented as mean ± *SEM* from three independent experiments, and each experiment was performed in triplicate. *p < 0.05; **p < 0.01. (e) Confocal immunofluorescence showing changes in the claudin‐5 (red) and ZO‐1 (green) expression especially in the cell–cell contact site at 72 h post‐exosome treatment. The experiment was repeated in triplicate. Magnification: 200×. Scale bar = 20 μm. (F) The in vitro BBB model of FITC–dextran permeability in cultured ECs subjected to the transfection with miR‐195 mimic (miR‐Exo), miR‐195 anti‐sense (anti‐miR‐Exo), or negative control (NC‐Exo) miRNA. Data are presented as the mean ± *SEM* from three independent experiments. *p < 0.05; **p < 0.01; ***p < 0.001 versus the corresponding time point of NC‐Exo

To investigate the relationship between miR‐195 and BBB integrity, we first created a general miR‐195a knockout (KO) mouse in this study. Notably, a mouse has two miR‐195 genes (miR‐195a and miR‐195b) and only miR‐195a gene is KO in our mouse model. miR‐195 levels are higher in the brain than several internal organs (Ludwig et al., 2016). miR‐195 expression level was significantly reduced by 25%–50% in the brain and 50%–75% in other organs of our miR‐195a KO mice (Figure S1A‐S1B). Brain MRI images showed significant BBB leakage in miR‐195a KO mice, indicating a relationship between miR‐195 reduction and BBB breakdown regardless of age (Figure 1a). In general, the average BBB permeability increased with age in the WT mice (Figure 1a), which is also consistent with previous studies (Goodall et al., 2018). Two old wild‐type (WT) mice at age of 24 months still had good BBB integrity in MRI image, and these two mice also had better performance in memory tests (Morris water maze) than other mice of the same age. Gross examination of Evans Blue extravasation in the whole brain (Figure 1b) and the quantification analysis of extravasation demonstrated that miR‐195a KO mice had an increase in BBB leakage by threefold when compared to the WT littermates (Figure S1C). Furthermore, histological examination of cerebral vessels of miR‐195a KO mice revealed increased albumin leak across BBB to the brain parenchyma, which provides another line of evidence to support the role of miR‐195 in BBB integrity (Figure 1b). Moreover, the miR‐195 levels in the total brain decreased in an age‐dependent manner in the WT mice at ages of 4, 13, 15, 18, and 25 months old (Figure S1D). These results suggested that miR‐195 is likely to affect BBB integrity and contribute to age‐dependent BBB leakage.

To further demonstrate that a low level of cerebral miR‐195 directly causes BBB disruption, we compared BBB permeability among WT, brain‐specific miR‐195a KO mice (*Ctsk*‐*Cre*;*miR*‐*195a^fl^* ^/^ *^fl^*), and miR‐195a KO mice. Both brain‐specific KO mice and miR‐195a KO mice presented higher BBB permeability than the age‐matched WT (Figure 1c). Moreover, both brain‐specific KO mice and miR‐195a KO mice showed similar levels of BBB leakage (Figure 1c), suggesting that BBB breakdown is primarily by the loss of cerebral miR‐195.

### 2.2 miR‐195‐enriched astrocytes secrete exosomes to improve BBB integrity

We compared the miR‐195 levels between astrocytes and endothelial cells (ECs) which are the two major cells comprising BBB. miR‐195 levels were measured in both human and mouse astrocytes and ECs including the following five cell lines: human iPSC‐derived astrocyte, human iPSC‐derived EC, human umbilical vein endothelial cell (HUVEC), mouse cerebral endothelial cell line (b.End3 cells), and mouse astrocyte cell line (ALT cells). miR‐195 levels were consistently higher in astrocytes than ECs by approximately fivefold across all these five cell lines (Figure 1d). Given that BBB leakage is directly related to EC function, we hypothesized that miR‐195 or miR‐195‐regulated molecules can be transported from astrocytes to ECs. If so, exosomes may be a cargo for miR‐195 transportation between astrocytes and ECs.

To test our hypothesis, exosomes from miR‐195‐enriched or miR‐195‐depleted astrocytes were compared. We prepared three types of exosomes from astrocytes transfected with miR‐195 mimic (denoted as miR‐Exo), miR‐195 anti‐sense (denoted as anti‐miR‐Exo), and negative control (NC) miRNA (denoted as NC‐Exo). Using iTRAQ‐labeled proteomics, exosome‐specific markers including FLOT1, TSG101, CD9, and CD81 were identified in the exosome samples while non‐exosomal markers including GM130 and calnexin were not detected. Moreover, these three types of exosomes did not show any difference in morphology or number (Figure S1E–G). Compared with NC‐Exo, adding miR‐Exo into cultured ECs resulted in a significant increase in TJ proteins, claudin‐5, and ZO‐1, whereas anti‐miR‐Exo treatment decreased the expression of the same proteins in the cultured ECs (Figure S2A). Furthermore, miR‐Exo enriched the expression of the TJ proteins in the EC‐EC contact sites (Figure 1e). As expected, miR‐Exo‐treated ECs significantly improved BBB integrity in an in vitro model (Figure 1f).

### 2.3 miR‐Exo increases tight junction proteins by suppressing selective autophagy

**Figure 2:**
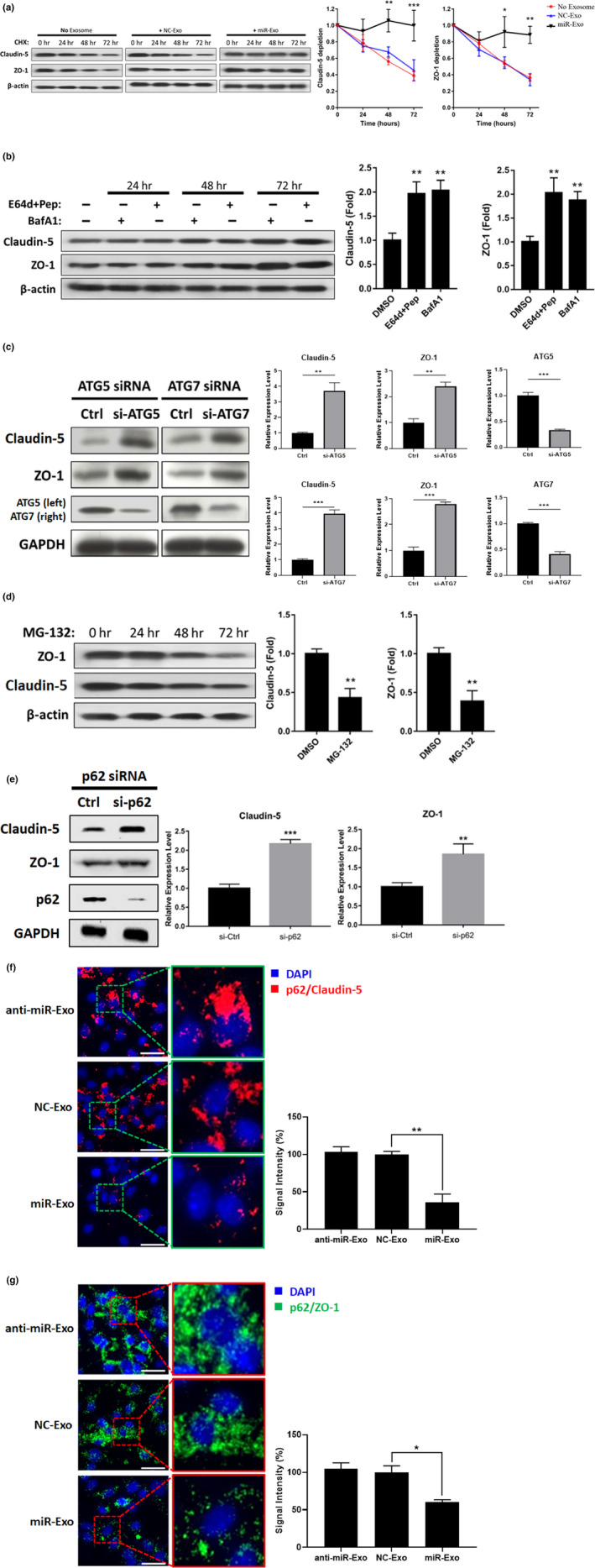
Selective autophagy of TJ proteins in a p62‐dependent manner. (a) Cycloheximide (CHX) was used to inhibit protein synthesis in exosome‐treated ECs. Stability of claudin‐5 and ZO‐1 proteins was increased by miR‐Exo. Representative Western blot and quantified data were from three independent experiments. (b) ECs were treated with different autophagy inhibitory agents, E64d + Pep and BafA1, up to 72 h, and TJ proteins were analyzed every 24 h. The quantified data indicated the change of proteins levels at 72 h. (c) Autophagosome components were knocked down by siRNA‐ATG5 or siRNA‐ATG7, and TJ proteins were analyzed at 72 h. Quantified data show the change of the protein levels at 72 h post‐si‐Ctrl or post‐siRNA treatment. (d) ECs were treated with the proteasome inhibitor MG‐132 up to 72 h, and TJ proteins were measured every 24 h. Quantified data show the change of the protein levels at 72‐h treatment. (e) Autophagic adaptor p62 was knockdown by siRNA, and TJ proteins were analyzed at 72 h. (f, g) p62/TJ complex was detected by proximity ligation assay (PLA) in exosome‐treated ECs at 24 h. The signal intensities, respectively, represent the amount of p62/claudin‐5 (red) and p62/ZO‐1 (green) complex in the ECs. The PLA results in the left and the quantitative data in the right. Data a–g are presented as mean ± *SEM* from three independent experiments. *p < 0.05; **p < 0.01; ***p < 0.001

Since an increase in TJ proteins was observed in miR‐Exo‐treated ECs, we then hypothesized that miR‐Exo might affect TJ metabolism by increasing expression or decreasing catabolism. Our experiments showed that miR‐Exo increased the abundance of the TJ proteins, but did not affect TJ mRNA levels (Figure S2B,C), which implied miR‐Exo may improve the stability of TJ proteins. Therefore, cycloheximide (CHX) was used to inhibit protein synthesis in ECs, and the data showed that miR‐Exo treatment substantially improved TJ protein stability (Figure 2a). Two major pathways are responsible for protein degradation: autophagy–lysosome and ubiquitin–proteasome pathways. The levels of TJ proteins were increased when autophagy was blocked by bafilomycin A1, a V‐ATP inhibitor (Figure 2b). Similarly, E64d and pepstatin suppressing autophagy by the inhibition of lysosomal protease also caused an increase in TJ proteins (Figure 2b). Our third approach was to knock down the autophagy components, ATG5 or ATG7, by siRNA, and again, TJ protein levels were increased (Figure 2c). On the contrary, inhibition of ubiquitin–proteasome pathway paradoxically reduced TJ protein abundance (Figure 2d). The reason for this opposite finding is because the inhibition of the ubiquitin–proteasome pathway can activate compensatory autophagy that exacerbates TJ protein degradation (Ji & Kwon, 2017). All these findings suggested that a suppression of the autophagy–lysosome pathway rather than the ubiquitin–proteasome pathway plays a primary role to upregulate TJ protein levels.

Given that TJ proteins are the targets for autophagic degradation, we next explored whether miR‐Exo increases TJ expression by inhibiting general autophagy. In fact, our data indicated that miR‐Exo treatment improved autophagic efficiency because Beclin‐1 and the ratio of LC3‐II/I were increased and p62 was reduced in ECs (Figure S2D). Obviously, the result from this experiment does not explain the above findings where suppression of autophagy increased TJ proteins leading to a decrease in BBB leakage. Accordingly, we speculated that miR‐Exo may suppress selective autophagy. To test this hypothesis, selective autophagy was suppressed by depleting autophagic adaptor p62, which subsequently led to an increase in TJ proteins (Figure 2e). The PLA and Co‐IP experiments further confirmed the formation of TJ‐p62 complex which facilitated TJ degradation (PLA data in Figure 2f,g; co‐IP data in Figure S2E,F). These experiments directly demonstrated that TJ proteins can be recognized by p62 and then degraded via selective autophagy resulting in BBB leakage. The treatment of miR‐Exo to ECs reduced TJ‐p62 complex, which urges us to search for the key component in miR‐Exo that affects selective autophagy.

### 2.4 Identification of thrombospondin‐1 (TSP1) as a key regulator for selective autophagy and BBB function

We next searched for any major exosomal proteins that are involved in the suppression of selective autophagy. iTRAQ‐labeled proteomics was used to identify differentially expressed proteins (DEPs) between miR‐Exo and NC‐Exo. In addition, we used the bioinformatics algorithm, Ingenuity Pathway Analysis (IPA), to list known autophagy‐related proteins. Three proteins were indicated as DEP and also revealed by the IPA (Figure S3A), which were Ras‐related protein Rap‐1b, transforming protein RhoA, and thrombospondin‐1 (TSP1). Among these three proteins, TSP1 had the highest level and its high abundance was subsequently confirmed by Western blot (Figure S3B). Although TSP1 has been reported to mediate autophagy in a few cell types (Kalas et al., 2013; Torres, Gubbiotti, & Iozzo, 2017), its role in selective autophagy has not been documented. Therefore, we selected TSP1 to further study its potential effect on TJ proteins and BBB function.

### 2.5 TSP1 increases BBB leakage

**Figure 3:**
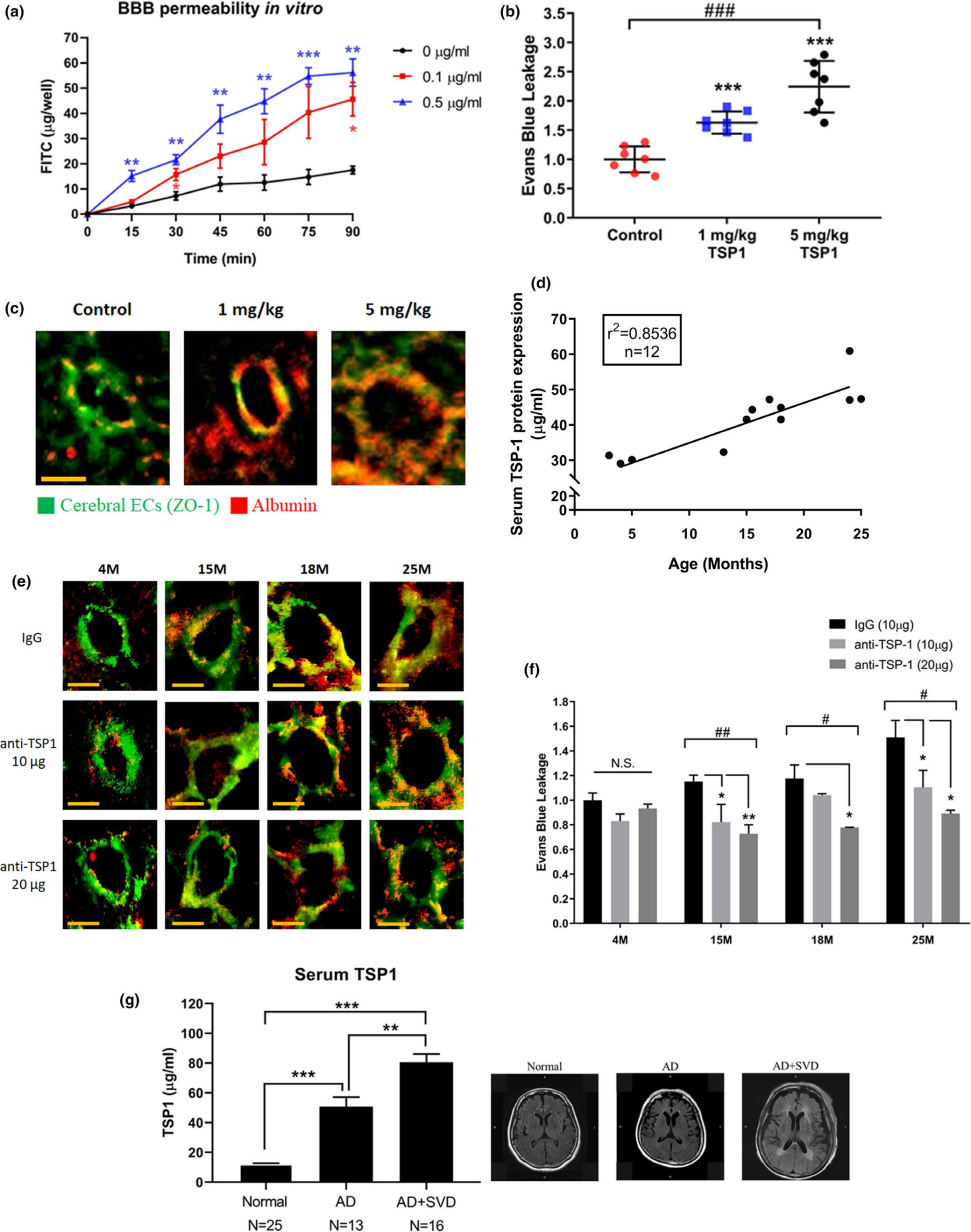
Serum TSP1 levels increased with age and led to BBB disruption. (a) TSP1 treatment exacerbated FITC–dextran leakage in an in vitro model. The corresponding time points in the untreated cells were used as the reference. (b) TSP1 was IV injected to mice, and BBB permeability was determined by extravasation of Evans blue on day 5. *n* = 7/group. (c) Extravasation of serum albumin (red) in TSP1 IV injected mice. Cerebral ECs were stained for ZO‐1 (green). Magnification: 200×. Scale bar = 20 μm. (d) Serum TSP1 levels measured by ELISA increase with age in C57BL/6 mice. See also Figure S3D. (e) IV injection of TSP1 antibody to neutralize serum TSP1. The histology analysis was used to evaluate BBB leakage on day 5. Extravasation of serum albumin (red) and cerebral ECs (green), *n* = 3/group. (f) Evans blue assay was used to quantify the effect of TSP1 antibody on BBB leakage. TSP1 antibody was IV injected to neutralize serum TSP1, and Evans blue assay was performed on day 5. *n* = 3/group. (g) Serum TSP1 levels in patients with and without small vessel disease (SVD) were measured by ELISA. The representative MRI images of patients are shown in the right; see also figure S3E. Normal, healthy control; AD, Alzheimer's disease; SVD, small vessel disease. Data are presented as the mean ± *SEM*. *p < 0.05; **p < 0.01; ***p < 0.001 using *t* test. #*p* < 0.05; ##*p* < 0.01; ###*p* < 0.001 for multiple group comparison using one‐way ANOVA

To confirm the role of TSP1 in the BBB function, we conducted a series of studies. First, we demonstrated that the effect of TSP1 on the BBB permeability using in vitro and in vivo models. For the in vitro BBB model, a Transwell assay showed that TSP1 dose‐dependently impaired EC integrity by exacerbating FITC–dextran leakage (Figure 3a). For the in vivo study, recombinant TSP1 protein was intravenously (IV) injected to the mice and BBB leakage was dose‐dependently increased as demonstrated by the extravasation of Evans blue in the brains (Figure 3b). Secondly, the effect of TSP1 on BBB leakage was supported by the histological images that showed EC disruption and albumin extravasation (Figure 3c). To test whether cerebral TSP1 also affects the BBB function, the second in vivo model was used where TSP1 was intracerebroventricularly (ICV) injected into the mouse brain. Again, the results supported that cerebral TSP1 also increased BBB leakage (Figure S3C). Taken together, the data from in vitro and in vivo models confirmed that TSP1 increased BBB leakage, which indicated that the alteration of TSP1 levels may be a major factor to explain the effect of miR‐Exo on TJ proteins as shown in Figure 1e,f.

Because BBB integrity decreases with age, we speculated whether the circulating TSP1 levels increase with age. If so, the age‐induced BBB leakage can be rescued by the TSP1 antibody. Indeed, our data showed that the circulating TSP1 levels in C57BL/6 mice increased with age with the correlation coefficient of 0.85 (*p* = 0.025) (Figure 3d and Figure S3D). The IV injected TSP1‐neutralizing antibody reduced BBB leakage in mice of different ages (Figure 3e), and the therapeutic effect was more significant in aged mice. In addition, we used the Evans blue assay to quantify the effect of TSP1 neutralizing antibody (Figure 3f). Again, the treatments reduced BBB leakage in a dose‐dependent manner.

To further demonstrate the importance of the TSP1 effect on cerebrovascular integrity, we compared the serum TSP1 levels in Alzheimer's dementia (AD) patients with or without small vessel disease and age‐matched healthy subjects. The diagnosis of BBB integrity was based on MRI images of these subjects (Figure 3g and Figure S3E). The data clearly showed that TSP1 levels were significantly higher in AD with small vessel disease than AD without small vessel disease (*p* = 0.002). TSP1 levels in the normal subjects were substantially lower than AD subjects regardless of small vessel disease (*p* < 0.0001).

Taken together, our results indicated that TSP1 has a causal relationship role in BBB leakage. More importantly, our results suggest TSP1 may be a drug target for BBB leakage‐induced cognitive impairment.

### 2.6 TSP1 decreases tight junction proteins by activating selective autophagy

**Figure 4:**
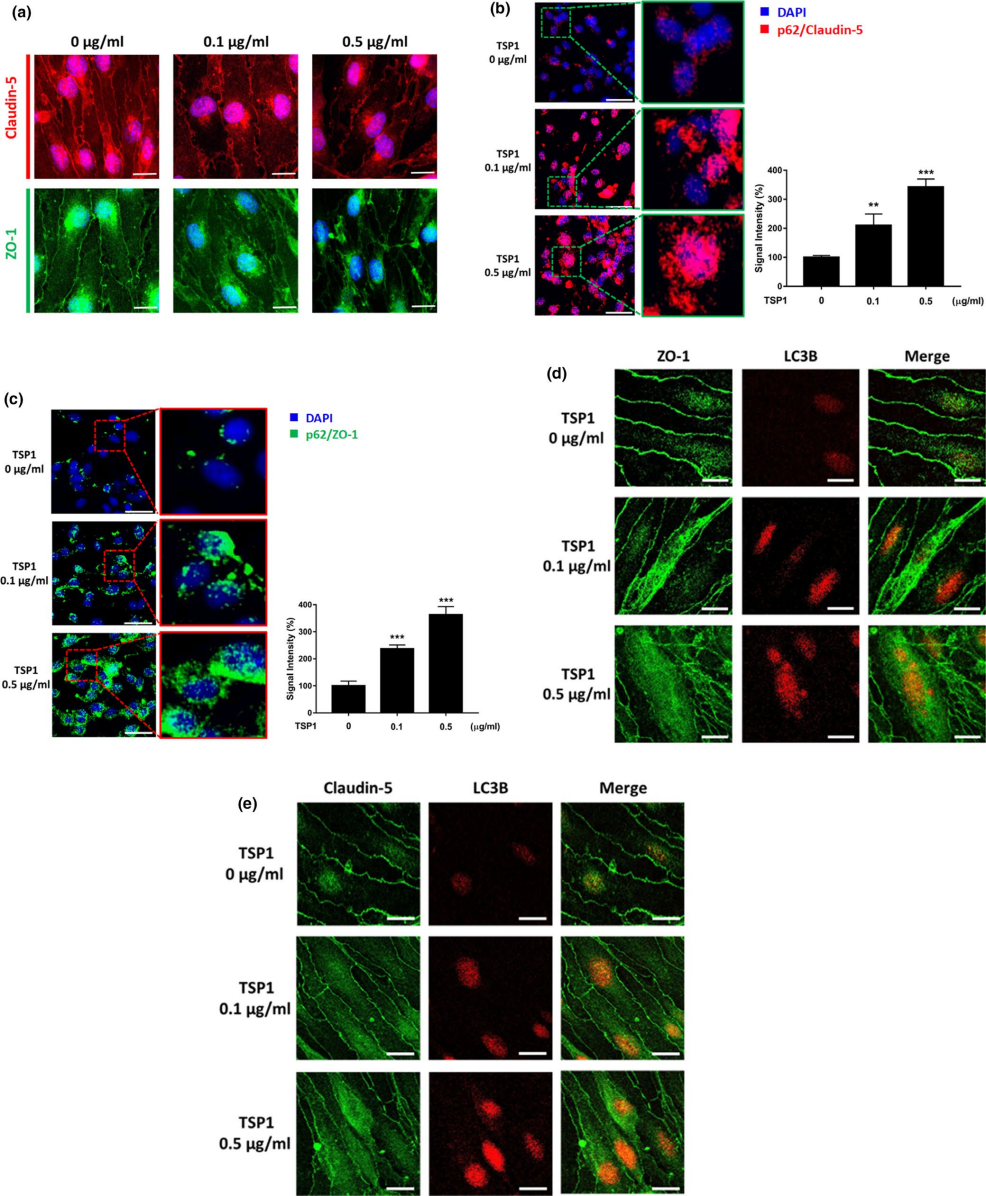
TSP1‐induced selective autophagy to catabolize TJ proteins. (a) Confocal immunofluorescence analysis showed TSP1 diminished claudin‐5 (red) and ZO‐1 (green) expression in the cytoplasm of ECs and EC‐EC contact sites at 72 h post‐TSP1 treatment. Magnification: 200×. Scale bar = 20 μm. (b, c) p62‐TJ protein interactions were determined on TSP1‐treated ECs by the proximity ligation assay (PLA). PLA was performed in the TSP1‐treated ECs for 24 h. The red and green signals, respectively, represent p62/claudin‐5 and p62/ZO‐1 interaction in ECs. The PLA results are shown in the left and the quantitative data in the right. See also Figure S4B,C. (d, e) The confocal images show TSP1‐induced translocation of TJ proteins in ECs at 24 h. The TJ proteins were labeled with antibodies, and autophagosome was labeled with LC3B. In the left panel, the immunofluorescent images show that TJ proteins were redistributed from cell–cell contact sites to the cytoplasm in a dose‐dependent manner. In the middle and right panels, the cytoplasmic TJ proteins were co‐localized with LC3B, suggesting that TSP1 induces internalization of TJ proteins into autophagosomes. Data are presented as mean ± *SEM* from three independent experiments. **p < 0.01; ***p < 0.001

Given the above convincing data, we explored whether TSP1 could decrease TJ proteins and regulate selective autophagy. Based on the immunofluorescence analysis, TSP1 indeed decreased claudin‐5 and ZO‐1 protein levels in the cytoplasm of ECs (Figure 4a and Figure S4A) and the EC‐EC contact sites (Figure 4a). Next, we tested the role of TSP1 in two critical steps of selective autophagy, which were formation of autophagic adaptor–cargo complex (p62‐TJ complex) and uptake of cargo proteins (TJ proteins) by autophagosomes. To examine the effect of TSP1 on p62‐TJ complex formation, we conducted two experiments. First, the PLA assay was used to investigate a direct link between TJ proteins and p62, and the results revealed that TSP1 increased p62‐TJ complexes (Figure 4b,c). Secondly, the results of co‐immunoprecipitation (Co‐IP) assay also suggested that TSP1 increased the formation of p62‐TJ complexes (Figure S4B,C). We next investigated whether TSP1 could induce uptake of TJ proteins to autophagosomes. By labeling LC3B on autophagosome, our confocal images indicated an increase in uptake of TJ proteins given that TSP1 promoted TJ protein trafficking from cell membrane toward autophagosomes. Figure 4d,e reveal that TSP1 caused redistribution of TJ proteins from the cell membrane to the cytoplasm and increased the co‐localization of TJ proteins and LC3B. The above findings are consistent with our hypothesis for the effect of TSP1 on selective autophagy.

In addition, we assessed the effect of TSP1 on general autophagy by Western blot. Beclin‐1 and LC3‐II/I ratios were decreased and p62 was augmented in TSP1‐treated ECs, suggesting the detrimental effect of TSP1 on general autophagy (Figure S4D). It has been documented that selective autophagy could be activated even if general autophagy was impaired in neurodegenerative diseases (Lim et al., 2015). Moreover, the data on impaired general autophagy further support our hypothesis that TSP1‐induced TJ degradation is primarily due to action of selective autophagy rather than general autophagy.

Taken together, these results suggested that TSP1 increased TJ degradation via an increase in selective autophagy, whereas TSP1 disrupted general autophagy. Furthermore, these results are in concert with those from the miR‐Exo experiments (Figure 2f,g), which support TSP1 as a key factor for astrocyte–EC cross talk.

### 2.7 Mir‐195 suppresses TSP1 synthesis and secretion

**Figure 5:**
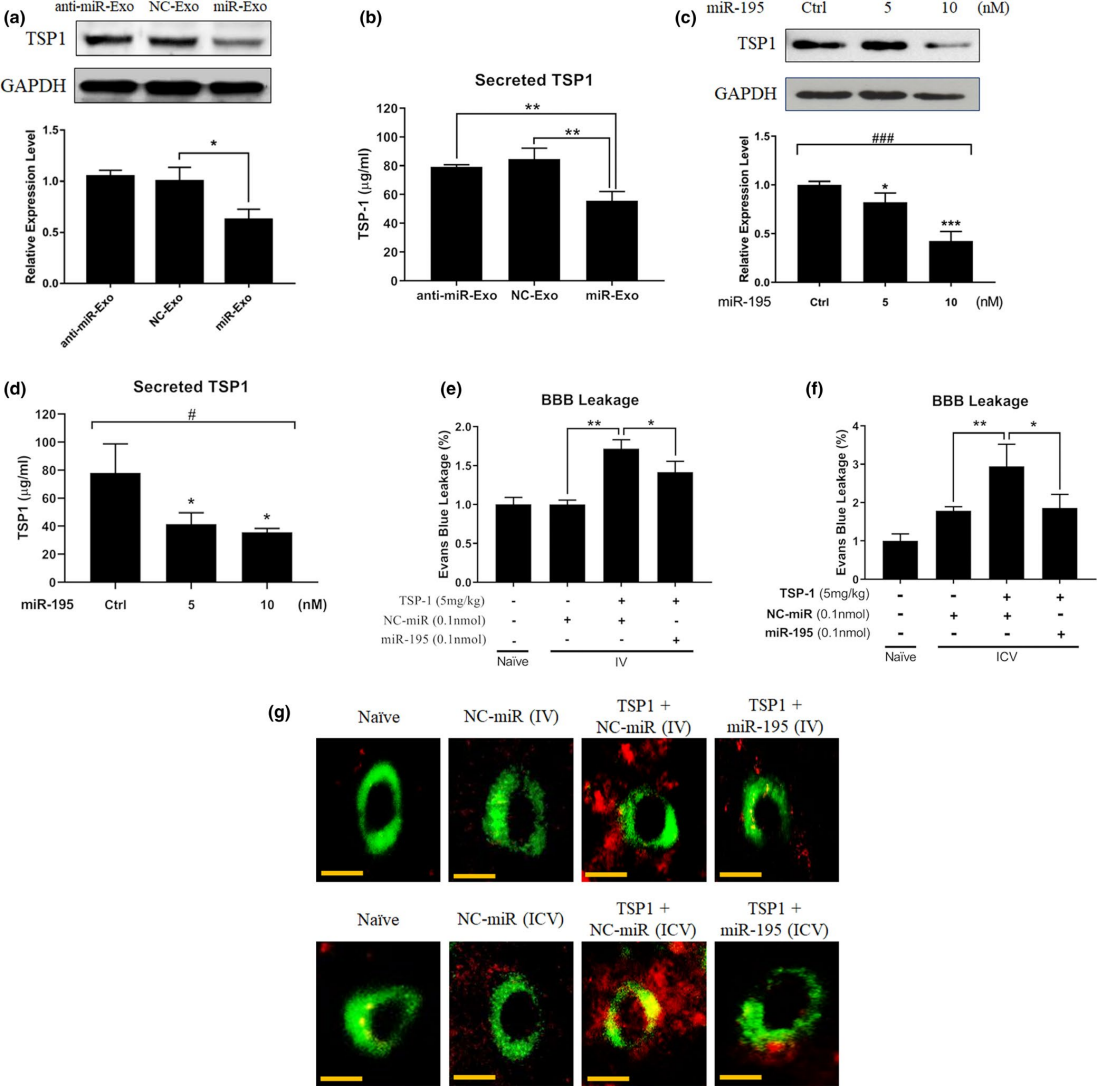
miR‐195 reversed TSP1‐induced BBB leakage. (a) Western blot shows TSP1 levels at 72 h post‐exosome treatment. (b) Secreted TSP1 in the cultured medium of ECs at 72 h post‐exosome treatment. (c) miR‐195 dose‐dependently inhibits TSP1 protein levels in ECs at 72 h after miR‐195 transfection. (d) Secreted TSP1 levels in cell culture medium at 72 h post‐transfection of miR‐195. All quantitative data are presented as mean ± *SEM* from three independent experiments. *p < 0.05; **p < 0.01 are calculated based on the reference group of NC‐Exo or control miRNA treatments. #*p* < 0.05; ###*p* < 0.001 for multiple group comparison using one‐way ANOVA. (e, f) miR‐195 reduces BBB permeability in TSP1‐treated mice. miR‐195 was given via IV (e) and ICV (f) injection. BBB permeability was measured by the Evans blue assay on day 5 post‐TSP1 and post‐miR‐195 injection. TSP1 and miR‐195 were injected on the same day. Data are presented as mean ± *SEM*, *n* = 3/group. *p < 0.05; **p < 0.01. (g) Extravasation of serum albumin (red) in TSP1‐ and miR‐195‐treated mice from figure e, f. Cerebral ECs were stained for ZO‐1 (green). Magnification: 200×. Scale bar = 20 μm. (*n* = 3/group)

We compared serum TSP1 levels between 4‐month‐old wild‐type and age‐matched miR‐195a KO mice. The result showed that serum TSP1 was 1.5‐fold higher in miR‐195a KO mice (*n* = 3/each group; *p* = 0.033). We then tested whether miR‐195 could be transported by exosomes from astrocytes to ECs. If so, we would like to know whether the exosome‐carried miR‐195 could suppress TSP1 in ECs. Our data showed that treating ECs with miR‐Exo, NC‐Exo, or anti‐miR‐Exo resulted in a corresponding change of the miR‐195 level in ECs (Figure S5A). Both intracellular TSP1 and secreted TSP1 levels were decreased after treated with miR‐Exo, indicating an inhibitory effect of miR‐195 on TSP1 expression in ECs (Western blot and ELISA in Figure 5a,b and mRNA in Figure S5B). Direct transfection of miR‐195 to ECs, which serves the positive control, resulted in the same findings (Western blot and ELISA in Figure 5c,d and mRNA in Figure S5C). In addition, miR‐195 increased general autophagy (Figure S5D) and upregulated TJ protein levels in cerebral ECs (Figure S5E). These results not only implied that miR‐195 protects BBB by inhibiting TSP1, but also partially explained the increased BBB leakage in miR‐195a KO mice as shown in Figure 1a,b.

To further confirm the effect of miR‐195 on TSP1‐induced BBB leakage, miR‐195 was injected in a rescue animal study. In TSP1‐treated mice, miR‐195 decreased BBB leakage no matter it was delivered via IV or ICV injection, although the ICV delivery resulted in a better effect (Figure 5e,f). Consistently, miR‐195 treatment significantly decreased albumin extravasation in the brain parenchyma (Figure 5g). The above data suggested that miR‐195 not only suppressed TSP1 expression and secretion but also rescued TSP1‐induced BBB leakage.

miR‐195 does not directly target human TSP1 mRNA based on the online bioinformatics databases (miRanda, TargetScan, and miRBase). Since TSP1 is upregulated by NF‐κB signaling (Lopez‐Dee, Pidcock, & Gutierrez, 2011) and our previous study found that miR‐195 negatively regulates NF‐κB pathway (Cheng et al., 2019), miR‐195 may suppress TSP1 expression by inhibiting NF‐κB.

### 2.8 TSP1 and CD36: mechanism of TSP1‐induced BBB leakage

CD36 is one of the endogenous receptors of TSP1, and it has been reported as a mediator of BBB integrity and autophagy inducer (Robertson et al., 2013; Sanjurjo et al., 2015). To verify the role of CD36 in TSP1‐induced BBB leakage, we knocked down CD36 in ECs and found that TSP1 no longer caused TJ redistribution and degradation (Figure S5F). Moreover, the binding between p62 and TJ proteins was also diminished after knocking down CD36, suggesting selective autophagy was activated via the TSP1/CD36 pathway (Figure S5G–I). These data, in conjunction with the foregoing findings, suggested that the axis of TSP1/CD36/selective autophagy of TJ proteins contributed to BBB leakage.

## 3 DISCUSSION

**Figure 6:**
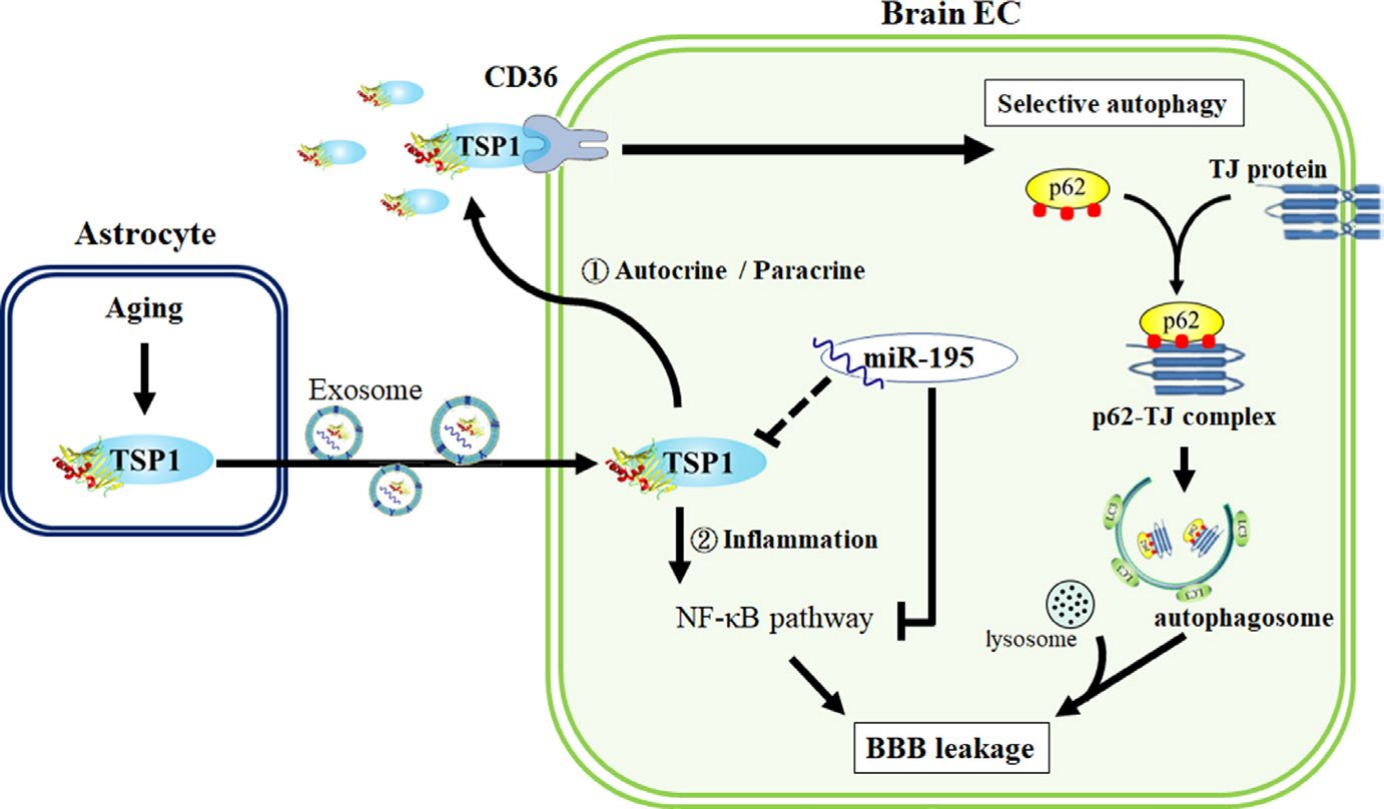
Schematic diagram shows the mechanism of TSP1‐induced BBB leakage and how miR‐195 suppresses the TSP1 signaling cascade

We previously showed that miR‐195 regulates vascular function (Cheng et al., 2019). In the present study, we first noticed that a low level of miR‐195 increased BBB leakage in both in vivo and in vitro studies. Through the analysis of astrocyte‐derived exosomes, we identified that miR‐195‐regulated TSP1 broke down BBB integrity. Our further investigation revealed a novel role of TSP1 in the degradation of TJ proteins by enhancing selective autophagy. After TSP1 bound to its receptor CD36, p62 tethered TJ proteins to a nascent autophagosome and then caused selective autophagy‐mediated protein degradation. Figure 6 schematically shows the mechanisms of TSP1‐mediated BBB leakage. The negative impact of TSP1 on BBB was further demonstrated in animals. Injection of TSP1 to the blood vessel or brain ventricle weakened BBB and led to extravasation of albumin in the brain parenchyma. Moreover, we showed that serum TSP1 levels were higher in patients of cerebral small vessel disease than controls, which further supports the role of TSP1 in BBB dysfunction. On the other hand, injection of TSP1 antibody or miR‐195 to the blood vessel or brain ventricle reduced BBB leakage in the animal model. Therefore, inhibition of TSP1 or its signaling may provide a potential therapeutic intervention to reduce BBB leakage.

Aging is a risk factor for neurodegenerative diseases and BBB dysfunction (Iadecola, 2017). Nevertheless, previous therapeutic strategies for neurodegenerative diseases primarily focused on rescuing neurons and repairing neuronal damage (Cai et al., 2017). Recently, the contribution of BBB integrity to the neurocentric view of brain diseases has been proposed (Nation et al., 2019). Our previous studies have indicated that miR‐195 exerts both neuroprotection and vasculoprotection in vascular injury and acute brain stroke (Cheng et al., 2019; Wang et al., 2012). The present study further elucidated the beneficial effect of miR‐195 on BBB integrity in aging animals. In addition, we identified miR‐195‐regulated TSP1 as a major molecule to degrade TJ proteins and increase BBB permeability through selective autophagy. This study not only offered prominent scientific insight into BBB integrity but also provided a novel therapeutic opportunity for aging‐related BBB leakage.

TSP1 is primarily released from platelets on activation, but can also be secreted from multiple cells, such as ECs and astrocytes in response to stress (LeBlanc & Kelm, 2017). We first discovered that the TSP1 levels in astrocyte‐derived exosomes were affected by miR‐195. Subsequently, we demonstrated that both serum and cerebral TSP1 increased BBB permeability. Increased BBB permeability can be demonstrated by white matter hyperintensities in the brain MRI image (Wardlaw et al., 2013). BBB leakage can incur in various clinical presentations. Patients with cerebral small vessel disease defined as consecutive lacunar stroke and mild vascular cognitive impairment were found to have BBB leakage based on structural brain MRI and DCE (dynamic contrast‐enhanced)‐MRI (Zhang et al., 2017). Accordingly, we measured serum TSP1 in patients with small vessel diseases to test for the association between TSP1 levels and BBB leakage in humans. Three types of human subjects were recruited: normal subjects without white matter hyperintensity in MRI, AD without small vessel disease, and AD with small vessel disease. The results showed that serum TSP1 levels were higher in subjects with BBB leakage in human samples, which supported the detrimental effect of TSP1 on human BBB breakdown.

A research group reported that air pollutant‐induced inflammation upregulated TSP1 that led to BBB disruption via CD36 membrane receptor (Aragon et al., 2017). Based on their earlier study (Robertson et al., 2013) where CD36 was shown to exacerbate vascular dysfunction by interacting with pollution‐induced molecules, this research group proposed and then demonstrated pollutant‐induced TSP1 as a contributor to BBB permeability (Aragon et al., 2017). On the other hand, we identified TSP1 based on the combination between proteomic analysis of astrocyte‐derived exosomes and bioinformatics analysis of autophagy‐related proteins. We further elucidated a distinct and a novel mechanism to explain how TSP1 contributes to age‐dependent BBB impairment. In addition, we showed that TSP1‐induced BBB damage can be rescued by exogenous miR‐195. The present study also found that serum TSP1 levels increased with age and TSP1 may serve as a biomarker for BBB permeability. Given that BBB leakage is a risk for several neurodegenerative diseases (Sweeney, Sagare, & Zlokovic, 2018), further studies are warranted to confirm the usefulness of TSP1 in predicting neurodegeneration. Targeting TSP1 or its signaling pathway including miR‐195 and CD36 may be a potential therapeutic strategy to slow down age‐dependent neurological diseases.

TSP1 is a ligand of multiple endothelial surface receptors, including tumor necrosis factor receptor 1 (TNF‐R1), CD47, and CD36 (Resovi, Pinessi, Chiorino, & Taraboletti, 2014). We demonstrated that selective autophagy activated by TSP1/CD36 is one major cause of BBB leakage. However, we cannot rule out the possibility of the involvement of other TSP1 signaling in BBB integrity. For example, TSP1/TNF‐R1 interaction induces apoptosis of cerebral ECs (Rege et al., 2009) and TSP1/CD47 signaling drives endothelial senescence (Gao, Chen, Gao, Zheng, & Yang, 2016). Thus, TSP1 is likely to activate multiple signal transductions to induce BBB disruption. The present study augments that TSP1 and its receptors are potential targets for the improvement of BBB integrity and for the prevention of neurodegenerative diseases.

Our team has found that miR‐195 has anti‐apoptotic, anti‐inflammatory, and neurovascular protective effects (Cheng et al., 2019). In the present study, we further demonstrated that miR‐195 regulates TSP1 and general autophagy signaling. A low miR‐195 level in the brain was reported to cause cognitive impairment in the animal model of chronic brain hypoperfusion (Ai et al., 2013). Several studies have demonstrated the beneficial effect of miR‐195 on the cognitive function, which was due to the suppression of APP, BACE1 expression (Ai et al., 2013), and tau phosphorylation (Sun et al., 2015). Although the current study did not directly measure the cognitive function, vascular dementia has been attributed to BBB disruption (Sweeney et al., 2018). Our data showed that miR‐195 has a protective effect on BBB integrity, which can be partially explained by reducing degradation of TJ proteins through the suppression of selective autophagy. Moreover, the supplement of exogenous miR‐195 rescued TSP1‐caused BBB disruption and albumin extravasation, highlighting a therapeutic potential of miR‐195 in neurovascular diseases.

General autophagy has been traditionally regarded as a nonselective degradation process (Kim et al., 2018) and has a profound effect on BBB functions by regulating the homeostasis of the TJ proteins (Yang et al., 2019). Because the effect of miR‐195 can be tissue‐specific (Cheng et al., 2019), miR‐195 has been reported to have a negative or a positive effect on general autophagy depending on the tested cell types (Hou, Cheng, Wang, Wang, & Wu, 2019; Mo, Zhang, & Yang, 2016; Shi et al., 2013). Here, we demonstrated that miR‐195 improved the efficiency of general autophagy in cerebral ECs. Recent studies have suggested that autophagy can be highly selective. Selective autophagy refers to selective degradation by promoting interaction between the adaptor proteins and specific substrates (Yamamoto & Yue, 2014), which may play a critical role in neurodegenerative diseases and aging (Anding & Baehrecke, 2017). However, the role of selective autophagy in TJ metabolism and BBB integrity remains largely unclear.

In conclusion, the present study not only demonstrated the important role of miR‐195 in maintaining BBB integrity, but also illustrated that miR‐195‐regulated TSP1 increased TJ protein degradation and weaken BBB integrity by selective autophagy. Our human data suggested that circulating TSP1 can be used as a therapeutic target and biomarker of BBB leakage. The study implies that a sufficient level of miR‐195 and a low level of TSP1 can suppress selective autophagy of TJ proteins, which is important to maintain BBB function and to reduce the risk for neurodegeneration.

## 4 EXPERIMENTAL PROCEDURES

### 4.1 miR‐195a knockout (KO), brain‐specific miR‐195a KO, wild‐type (WT), and aged mice

MiR‐195a KO mice on a C57BL/6 background were generated by the National Laboratory Animal Center, Taiwan. To generate brain‐specific miR‐195a KO mice, mice homozygous for floxed *miR*‐*195a* alleles (*miR*‐*195a^fl^* ^/^ *^fl^*) were crossed with those expressing Cre recombinase under the control of *Ctsk* promoter to generate *Ctsk*‐*Cre*;*miR*‐*195 ^fl^* ^/^ *^fl^* mice. Both *miR*‐*195a^fl^* ^/^ *^fl^* mice and *Ctsk*‐*Cre* mice were generated by the National Laboratory Animal Center, Taiwan. Genomic DNA isolated from tail biopsies was used to confirm the genotype of the mice. Cre recombinase is expressed in brain, eyes, heart, spleen, skeleton muscle, small intestine, appendix, and skin. Since the mice were used to study BBB integrity in the present study, *Ctsk*‐*Cre*;*miR*‐*195 ^fl^* ^/^ *^fl^* mice were denoted as brain‐specific KO mice in the present study.

### 4.2 Cell culture

Mouse astrocyte cell line ALT (BCRC60581) and mouse cerebral endothelial cells bEnd.3 (BCRC60515) were obtained from Bioresource Collection and Research Center, Taiwan. ALT and bEnd.3 cells were maintained in DMEM supplemented with 10% FBS (Invitrogen, Waltham, MA, USA), 1% penicillin and streptomycin (Biowest, Loire Valley, France), and 1% l‐glutamine (Invitrogen) in a humidified incubator under an atmosphere of 5% CO_2_ at 37 °C.

### 4.3 BBB permeability determined by the Evans blue assay

BBB disruption was assessed by measuring the amount of Evans blue extravasation. Briefly, mice were intravenously injected with 0.1 ml 4% Evans blue (Sigma‐Aldrich). After 60 min, the animals were perfused transcardially with phosphate‐buffered saline (PBS) to remove intravascular Evans Blue. The brains were homogenized in formamide (Sigma‐Aldrich) and incubated in the dark at 60°C for 24 h. The absorbance of eluted Evans blue dye in formamide solution was measured using a spectrophotometer at 610 nm and normalized to plasma levels.

### 4.4 BBB integrity measured by MRI

#### 4.4.1 Animals

Male C57BL/6 WT mice and the age‐ and sex‐matched miR‐195a KO mice were used for MRI tests. To perform MRI measurement, mice were tail‐vein‐injected with Resovist (5 mg/kg, Schering AG), a clinically approved superparamagnetic iron oxide (Fe_3_O_4_) developed for contrast‐enhanced magnetic resonance imaging (MRI) (Reimer & Balzer, 2003), under the anesthesia with isoflurane (Proane^®^, Aesica Queenborough, Lte.).

##### MR Data Acquisition

MRI experiments were performed with a 9.4 T horizontal‐bore animal MR scanning system (Biospec 94/20) equipped with a mouse‐imaging cryocoil (MRI CryoProbe). The imaging parameters used for the studies were as follows: field of view =19.2 × 12.8 × 6.4 mm^3^, matrix dimension = 384 × 256 × 128, voxel size =50 × 50 × 50 μm^3^, repetition time (TR) = 50 ms, echo time (TE) = 13.5 ms, slice thickness = 0.1 mm, bandwidth = 25 kHz, and total scan time = 27 min and 18 s. The large‐scale B0 inhomogeneity was minimized by region of interest (ROI)‐based shimming (provided with the system).

##### MR Data Processing

Multichannel MR images were reconstructed using MATLAB (The MathWorks) and then separated into magnitude and phase images. The magnitude images of the individual channels of the coil array were combined using the sum‐of‐squares method, and the phase images were assembled using complex summation. Subsequently, the combined magnitude and phase images were used for quantitative susceptibility mapping (QSM) reconstruction. For quantitative analysis of QSM images, images were processed using ImageJ to analyze the area of Resovist extravasation. The BBB integrity was determined by the difference of QSM quantified at 1 h to that at time 0.

### 4.5 FITC–dextran permeability assay

The permeability of in vitro BBB model was assessed using FITC–dextran fluorescein. The ECs were seeded onto 6‐well collagen‐coated Transwell inserts (0.4 μm pore size) (Millipore) at 5 × 10^5^ cells per well. ECs were grown to confluence to mimic the cell–cell environment in the vasculature, and ECs were subsequently treated with exosomes or TSP1 depends on experimental need. After 72 h, 50 μl of 5 mg/ml FITC–dextran (40 kDa, Sigma‐Aldrich) was added to the inner chamber with a final volume of 0.5 ml. To determine the permeability of the EC cellular barrier, aliquots of 50 μl from the outer chamber were taken every 15 min. The extravasated FITC–dextran was measured with excitation and emission wavelengths at 485 and 535 nm using a SpectraMax spectrophotometer.

### 4.6 Immunofluorescence (IF) analyses and proximity ligation assay (PLA)

Brain ECs were grown to confluence to mimic the cell–cell environment in the vasculature on coverslips for immunofluorescence (IF) or PLA. For both procedures, fixation and immunolabeling were performed in the same way according to standard protocols. Briefly, after respective treatment, the samples were fixed with 4% PFA and blocked in 5% bovine serum albumin (BSA) in PBS with 0.1% Triton X‐100 for 1 h at room temperature. For IF, the samples were incubated with ZO‐1 or claudin‐5 antibody in PBS with 5% BSA overnight at 4°C and incubated with appropriate secondary antibody (diluted 1:200 in PBS plus 0.01% Tween‐20 and 2% BSA). Secondary antibodies were conjugated by Alexa Fluor 488 or 647 (Invitrogen). For PLA, the samples were incubated with p62 (Abcam, ab56416), ZO‐1, and claudin‐5 antibodies were incubated overnight at 4°C. Proximity ligation was performed utilizing the Duolink^®^ In Situ Red/Green Starter Kit Mouse/Rabbit (Sigma‐Aldrich) according to the manufacturer's protocol. The oligonucleotides and antibody–nucleic acid conjugating reagents were provided by PLA kit (Sigma‐Aldrich, DUO92101). Both IF and PLA images were obtained by immunofluorescence confocal microscopy (Leica SP2/SP8X), and the images were analyzed with ImageJ software (NIH, MD).

### 4.7 iTRAQ‐Labeled proteomics

To test whether miR‐195 affected exosome component, iTRAQ was applied for the proteomic analysis of exosomes. NC‐Exo and miR‐Exo were collected by ultracentrifugation, and the following two steps were performed:

#### 4.7.1 8‐plex iTRAQ labeling

40 μg of protein samples was resuspended in 25 mM triethylammonium bicarbonate (TEABC, pH 8.5) and digested with trypsin at 37°C for 16 h. Digested peptide samples were labeled with 8‐plex iTRAQ reagents, and the reaction was quenched by adding equal volume of Milli‐Q water. After labeling, the samples were concentrated to dryness and dissolved with 0.1% FA and purified by Oasis^®^ HLB cartridges (Waters Corporation, USA).

#### 4.7.2 Nano LC‐MS/MS analysis

Nano LC‐MS/MS was performed with a nanoflow ultra‐performance liquid chromatography system (UltiMate 3000 RSLCnano System; Dionex) coupled to a hybrid quadrupole time‐of‐flight (Q‐TOF) mass spectrometer (maXis Impact; Bruker). After sample loading, the peptides were eluted from a trap column into an analytical column (Acclaim PepMap C18; Thermo Scientific) coupled to a nanoelectrospray ionization source on the Q‐TOF mass spectrometer. A gradient elution of 1% ACN (0.1% FA) to 40% ACN (0.1% FA) over 75 min was used at a flow rate of 300 nl/min for tryptic peptide separation. Sixteen precursors of charge +2, +3, and +4 from each TOF MS scan were dynamically selected and isolated for MS/MS fragment ion scanning. The selected precursors were then actively excluded for 0.4 min. The MS and MS/MS accumulation were set at 1 and 10 Hz, respectively.

### 4.8 Patient selection

The inclusion criteria for the study subjects recruited from Kaohsiung Medical University Hospital are (a) diagnosis with Alzheimer's disease according to diagnostic criteria described by the National Institute on Aging–Alzheimer's Association (NIA‐AA) (McKhann et al., 2011); (b) aged between 60 and 80 and; (c) no clinical history of stroke; and (d) ambulatory and able to undergo brain MRI. Among all subjects with AD, we defined the presence of cerebral small vessel disease if there are multiple infarcts but presented as silent stroke or severe white matter hyperintensity burden (Fazekas scale ≧ 2 on either periventricular area or deep white matter area) on brain MRI (Chen et al., 2016).

### 4.9 Animal studies

In the present study, BBB integrity was evaluated mainly by evaluating the amount of Evans blue and albumin extravasation. The high permeability of BBB in miR‐195 KO mice was also confirmed by MRI technique. In BBB leakage model, recombinant TSP1 was IV injected into 4‐month‐old WT mice and the severity of BBB disruption was evaluated on day 5 postinjection. For the in vivo TSP1 inhibition experiments, TSP1 antibodies were IV injected into 4‐, 15‐, 18‐, and 25‐month‐old C57BL/6 mice, and IgG‐injected mice were served as controls. Similarly, the effect of TSP1 antibodies on BBB permeability was assessed on day 5 postinjection.

The miR‐195 mimic or normal control microRNA (NC‐miR) was dissolved in Invivofectamine 3.0 reagent (Invitrogen) and subjected to TSP1‐treated mice by either IV or ICV injection. To conduct ICV injection, mice were anesthetized by isoflurane (1.2 ± 0.2%), and the head was secured in a stereotaxic apparatus (Stoelting, Wood Dale, IL, USA). Following the incision and exposing the skull, two small holes were drilled (±1.0 mm medial/lateral, ±0.5 mm anterior/posterior). A 33‐g needle was lowered into the ventricles (±2.4 mm dorsal/ventral), and microRNA (0.5 nmol per ventricle) was infused. The needle was removed slowly to avoid infuscate backflow. The skull was closed with bone wax and sutured. The effect of miR‐195 on BBB permeability was assessed on day 5 postinjection.

### 4.10 Study approval

Kaohsiung Medical University Hospital Institutional Review Board approved human sample collection with IRB number KMUHIRP‐SV(I)‐20150089. The Animal Care and Use Committee of China Medical University approved the animal experimental protocols (approval number CMUIACUC‐2017‐292), which strictly conforms to the NIH Guide for the Care and Use of Laboratory Animals (NIH Publication No. 85‐23, revised 1996).

### 4.11 Statistical analysis

All values in the text and figures are expressed as mean ± *SEM*. Statistical differences were evaluated using Student's *t* test. A *p* value less than 0.05 was considered to indicate statistical significance in all experiments. One‐way ANOVA was used for multiple group comparison, and a *p* value less than 0.05 was considered to indicate statistical significance. Analysis of the data and plotting of the figures were performed using Prism 7 software (GraphPad Software Inc., San Diego, CA, USA).

## CONFLICT OF INTEREST

The authors declare no competing interests.

## AUTHOR CONTRIBUTIONS

C.‐Y.C. designed studies, conducted the experiments, interpreted the data, and wrote the draft. Y.‐M.C. conducted the animal experiments. H.‐F.L. provided patient samples and data, interpreted the results, and wrote the manuscript. C.‐J.C. conducted the proteomic experiments. C.‐S.C. provided patient samples and data, interpreted the results, and wrote the manuscript. J.‐L.Y. designed studies and interpreted the data. J.Y.H.C. designed studies, interpreted the data, and wrote the manuscript. S.‐H.H.J. designed and supervised studies, interpreted the data, and wrote and proved the draft.

## Supporting information

Fig S1‐S5

## Data Availability

The data that support the findings of this study are available from the corresponding author upon reasonable request.
