# Supplementary material for: miR‐195 reduces age‐related blood–brain barrier leakage caused by thrombospondin‐1‐mediated selective autophagy
Source: Aging Cell. 2020 Oct 7;19(11):e13236. doi: 10.1111/acel.13236 (PMC7681043; doi:10.1111/acel.13236)
Supplement: Supplementary file 1 — Fig S1‐S5 [file ACEL-19-e13236-s001.docx]

**Supplemental Information and Figures**

**Materials and Methods**

**Supplemental Fig 1.** Expression level of miR-195 affects BBB permeability but had no effect on morphology and number of astrocyte-derived exosomes.

**Supplemental Fig 2.** miR-Exo increased TJ protein levels but had no effect on TJ mRNA.

**Supplemental Fig 3.** TSP1 is the key factor that induces BBB leakage.

**Supplemental Fig 4.** TSP1 suppresses TJ proteins expression by activating selective autophagy.

**Supplemental Fig 5.** miR-195-regulated TSP1 increased selective autophagy of TJ proteins via membrane receptor CD36.

**Materials and Methods**

**Reagents**

MiR-195 mimic, anti-miR-195 and negative control microRNA (NC-miR) were purchased from Ambion Inc. (Austin, TX, USA) with the sequence’s information: miR-195 mimic, 5’-UAGCAGCACAGAAAUAUUGGC-3’; anti-miR-195, 5’-GCCAATATTTCTGTGCTGCTA-3’; negative control sequence, 5’-AGUACUGCUUACGAUACGG-3’. SYBR^®^ Green PCR Master Mix, MultiScribe^®^ Reverse Transcriptase Kit, TaqMan^®^ miR-195 and U6 Assays were purchased from Applied Biosystems (Foster City, CA, USA). Protein synthesis inhibitor cycloheximide was purchased from Sigma-Aldrich (St. Louis, MO, USA) and both proteasome inhibitor MG-132 and autophagy inhibitor bafilomycin A1, E64d and Pepstatin were obtained from MedChemExpress^®^ (Monmouth Junction, NJ, USA). Recombinant mouse TSP1 was purchased from R&D Systems (Minneapolis, MN, USA) and TSP1 neutralizing antibody was purchased from Thermo Fisher Scientific Inc. (MA5-13398; Waltham, MA, USA). Unless otherwise specified, all other reagents were of analytical grade. Primary antibodies anti-Claudin-5 (ab15106, Abcam; Cambridge, MA, USA), anti-ZO1 (ab221547, Abcam), anti-GAPDH (5174, Cell Signaling; Beverly, MA, USA), anti-ATG5 (ab228668, Abcam), anti-ATG7 (ab53255, Abcam), anti-p62/SQSTM1 (ab56416, Abcam), anti-FLOT1 (sc-133153, Santa Cruz; Dallas, Texas, USA), anti-TSG101 (14497-1-AP, Proteintech; Rosemont, IL, USA), and anti-TSP1 (MA5-13398, Thermo Scientific) were used in western blot, PLA, co-IP and immunofluorescence experiments.

**MicroRNA transfection and exosome isolation from cell culture medium**

miR-195 mimic, anti-miR-195 and NC-miR were respectively transfected into cultured astrocytes by HiPerFect Transfection Reagent (QIAGEN; Hilden, Germany.) for 72 h. To isolate exosomes, the culture medium was first collected on ice and pre-cleared from cell debris by sequential centrifugation (1,500 ×g for 10 min, 4,500 ×g for 10 min, and 10,000 ×g for 30 min). Next, exosomes were obtained by ultracentrifugation at 100,000 × g for 4 h using SW 40 Ti rotor (Beckman-Coulter). The pelleted fractions were resuspended in PBS or DMEM depend on experimental needs.

**Exosome quantification and analysis**

Exosomes isolated from culture medium were resuspended in 1 ml of PBS. The number of particles and particle size were measured using a nanoparticle tracking analysis (NTA) device NanoSight LM10 coupled to a CCD camera and a laser emitting a 60-mW beam at 405 nm (Malvern, United Kingdom). Video acquisitions were performed in 5 records of 60 s using the following parameters: shutter = 604, gain = 100, and threshold = 10. At least 1,000 particles were tracked in each sample.

**Transmission electronic microscopy (TEM)**

For TEM analysis, isolated exosomes were resuspended in 2% paraformaldehyde and 0.125% glutaraldehyde and deposited onto formvar-carbon-coated electron microscopy grids (EMS, FCF200H-Cu). The grids were transferred to 1% glutaraldehyde for 5 min and transferred to distilled water for 2 min. To contrast the exosomes, grids were treated with a drop of uranyl-oxalate solution (4% uranyl acetate; 0.15 M oxalic acid, pH = 7) for 5 min and transferred to methyl-cellulose-UA solution (a mixture of 4% uranyl acetate and 2% methyl cellulose in a ratio of 100 μl:900 μl) for 10 min. The grids were then removed with stainless steel loops and excess fluid blotted gently on Whatman filter paper. Grids were left to dry and imaged under a JEOL 1200 EX II transmission electron microscope.

**RNA isolation and measurement of miRNA and mRNA levels**

Total RNA was extracted from cells using Trizol reagent. A quantitative real-time PCR analysis using cDNA from the cells and tissues was performed using the AB7900 real-time PCR system (Applied Biosystems) according to the manufacturer’s instructions. For miR-195 and U6 detection, cDNA was synthesized from TaqMan MicroRNA Assays. The ratio of each gene was normalized to the internal control (small RNA U6 or GAPDH), and the expression levels were quantified by employing the 2^-△△Ct^ relative quantification method. Primers used for q-RT-PCR: Mouse Claudin-5 “ 5′-GTTAAGGCACGGGTAGCACT-3′ ” and “ 5′-TACTTCTGTGACACCGGCAC-3′ ”, Mouse ZO-1 “ 5′- GCTAAGAGCACAGCAATGGA -3′ ” and “ 5′- GCATGTTCAACGTTATCCAT -3′ ”, Mouse TSP1 “ 5′- GCGTTGCCAGGCTCCGAGTT -3′ ” and “ 5′- GGTGCGCAGGCCCTTCAGTT -3′ ”, Mouse GAPDH “ 5′- GTGCAGTGCCAGCCTCGTCC -3′ ” and “ 5′- GCCACTGCAAATGGCAGCCC -3′ ”.

**Cell treatments and chemical inhibitors**

Chemical inhibitors were used to study the effect of miR-Exo on TJ protein metabolism. To investigate whether miR-Exo increased TJ protein content by promoting TJ protein synthesis, 50 nM cycloheximide (CHX, Sigma-Aldrich), a protein synthesis inhibitor, was introduced into exosome-treated ECs. To clarify the major pathway that was responsible for TJ protein degradation, ECs were treated with either proteasome inhibitor or autophagy inhibitor. First, ECs were treated with the proteasome inhibitor MG-132 (20 μM) to investigate the effect of ubiquitin-proteasome pathway on TJ proteins degradation. To further confirm the autophagy-lysosome pathway was the major pathway that responsible for TJ protein degradation, ECs were treated with distinct autophagy inhibitors: bafilomycin A1 (BafA1, 10 μM) or E64d and Pepstatin (E64d+Pep, 10 μM each). The contents of TJ proteins were monitored every 24 h by western blot.

**Enzyme-Linked immunosorbent assay (ELISA) for secreted TSP1 level**

Human TSP1 ELISA kit (CSB-E08763h; CUSABIO, TX, USA) or mouse TSP1 ELISA kit (CSB-E08765m; CUSABIO) were used to detect TSP1 levels in human serum, mouse serum and cell culture medium. Human serum was obtained from healthy subjects and Alzheimer’s disease (AD) patients with/without cerebrovascular disease (CVD). Mouse serum was collected from C57BL/6 mice of 4, 15, 18, and 25 months of age. For cell culture medium preparation, ECs were transfected with miR-195 or treated with exosomes for 72 h and the conditioned medium was collected for the measure of TSP1 levels according to the manufacturer’s protocol.

**siRNA transfection**

siRNAs were transfected into ECs at 50 nM using Lipofectamine RNAiMAX transfection reagents (Invitrogen) according to manufacturer instructions. The following siGENOME siRNA pools were used in this study: ATG5 (Dharmacon, M-064838-02-0005), ATG7 (Dharmacon, M-049953-02-0005), p62/SQSTM1 (Dharmacon, M-047628-01-0005), and CD36 (Dharmacon, M-062017-01-0005). The gene silencing effectiveness was determined by real-time PCR and western blot.

(1A)

(1B)


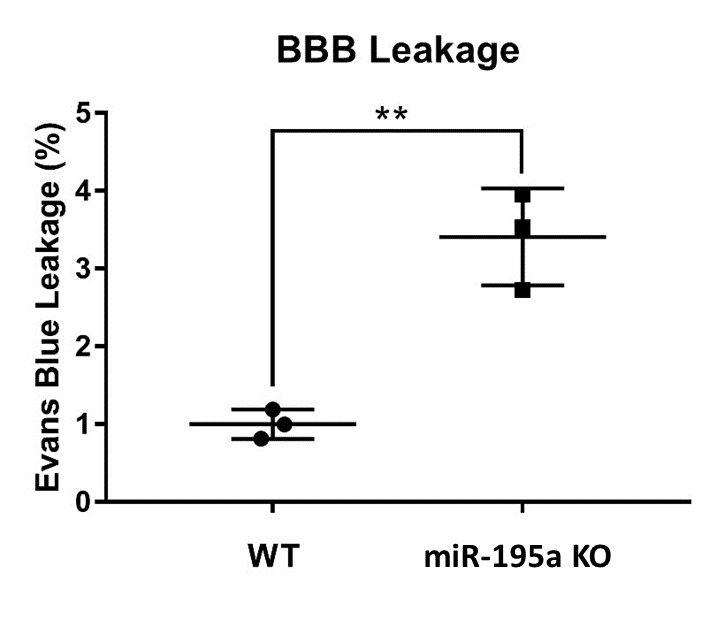
(1C)


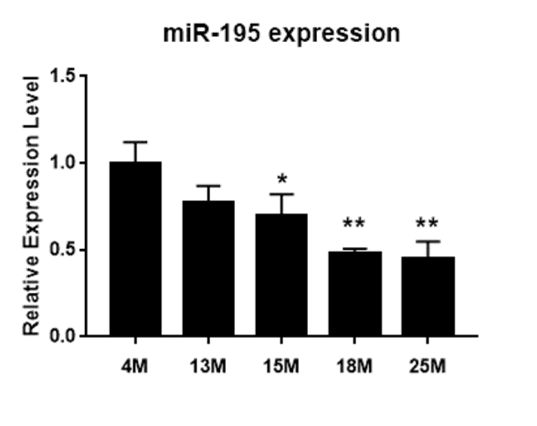
(1D)


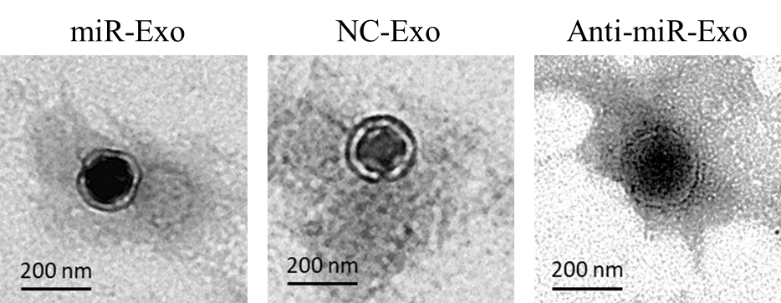
(1E)


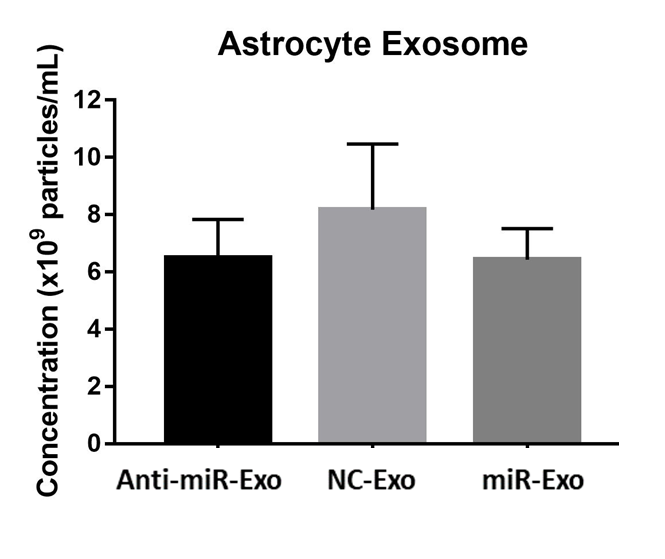
(1F)


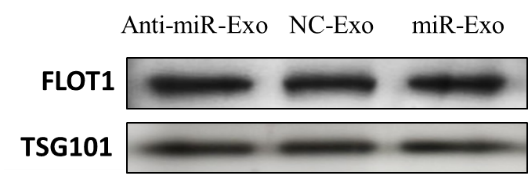
(1G)

**Supplemental Fig 1. Expression level of miR-195 affects BBB permeability but had no effect on morphology and number of astrocyte-derived exosomes.** (A-B) Expression levels of miR-195 were decreased by ~50% in central nervous system (A) and other organs (B) of miR-195a KO vs. age-matched WT mice. *n*=3 in each organ. (C) BBB permeability measured by Evans Blue assay in WT mice of 4-month-old and age-matched miR-195a KO mice (*n*=3/group). (D) An age-dependent decrease of miR-195 levels in the total brain (*n* = 3/group). **p* < 0.05; ***p* < 0.01 based on the data of 4-month-old mice. (E) Transmission electron microscopy (TEM) images of miR-Exo, NC-Exo, and anti-miR-Exo. Rounded-shaped structures with 150 nm average size were identiﬁed as exosomes. (F) The nanoparticle tracking analysis (NTA) software was used to measure the number of astrocyte-derived exosomes. Figure S1F showed the exosome number among miR-Exo, NC-Exo, and anti-miR-Exo from NTA measurements. Histograms are from analyses of at least 1000 individual tracks. Data are represented as mean ± SEM of three independent experiments. (G) Western blot analysis shows no significant change of exosome markers FLOT1 and TSG101. 15 μg of exosome samples were loaded for western blot analysis. Data are presented as mean ± SEM. **p*<0.05, ***p* < 0.01.


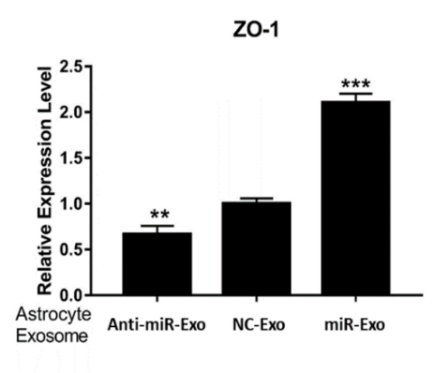
(2A)


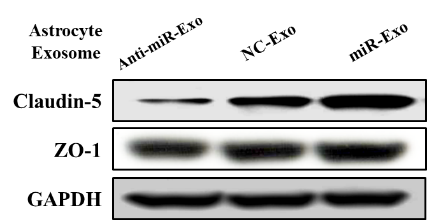

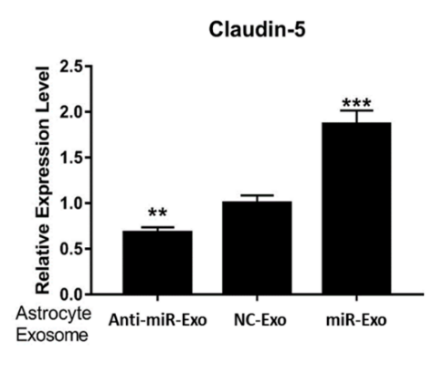


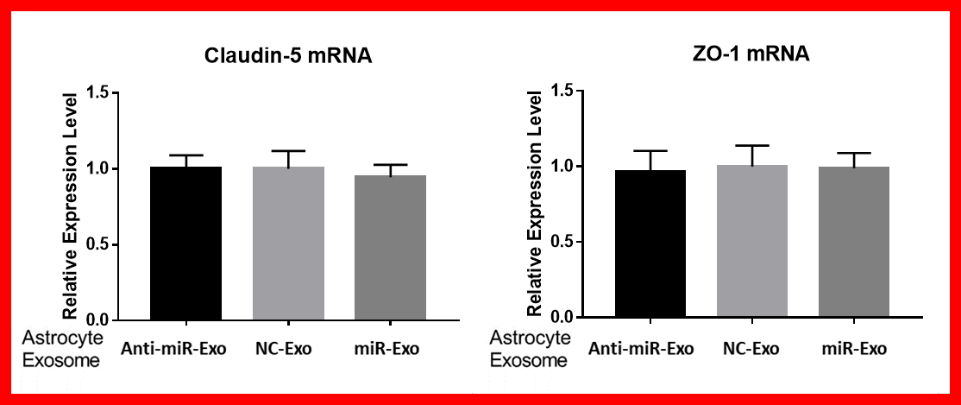

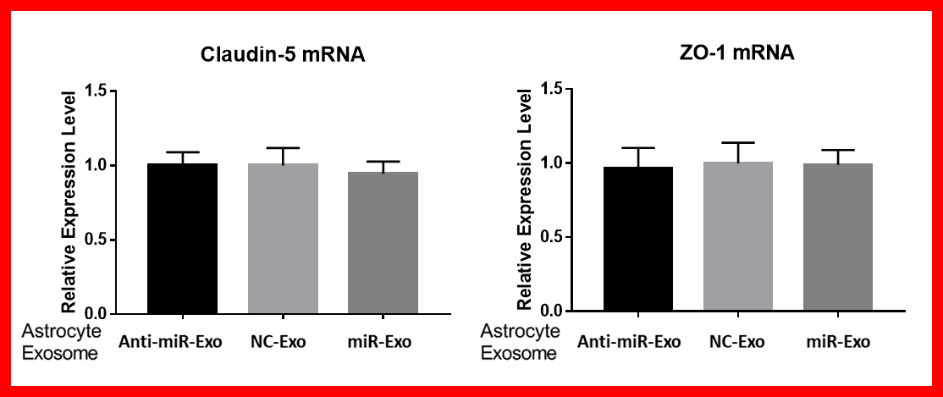
(2B) (2C)

(2D)


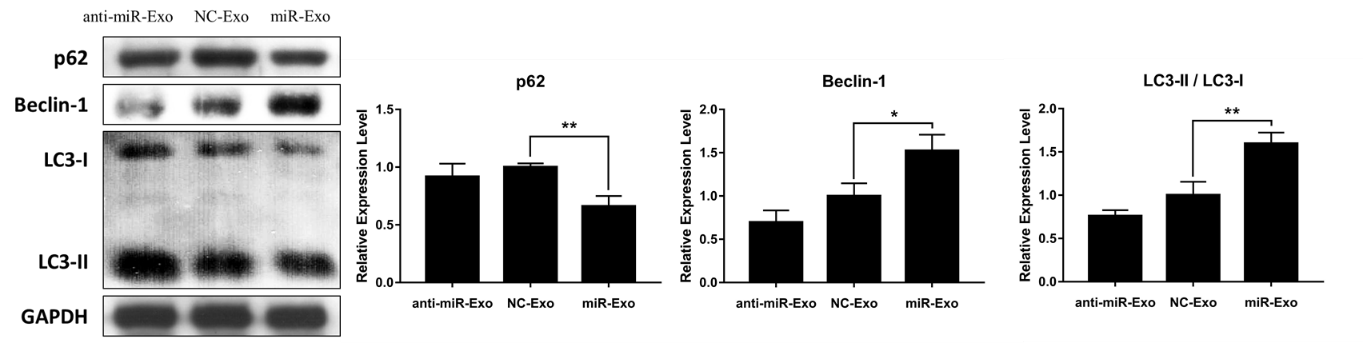


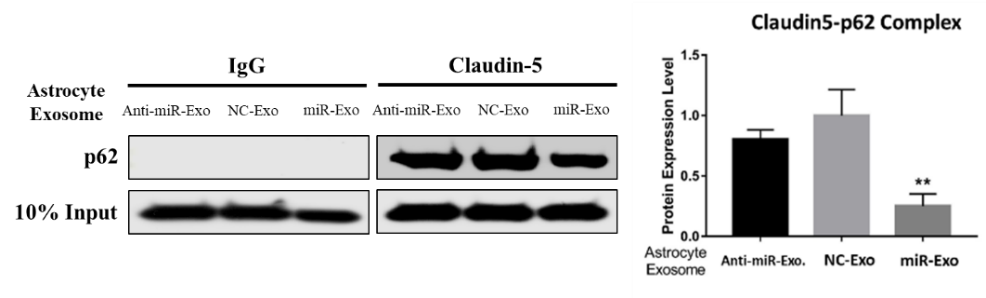
(2E)


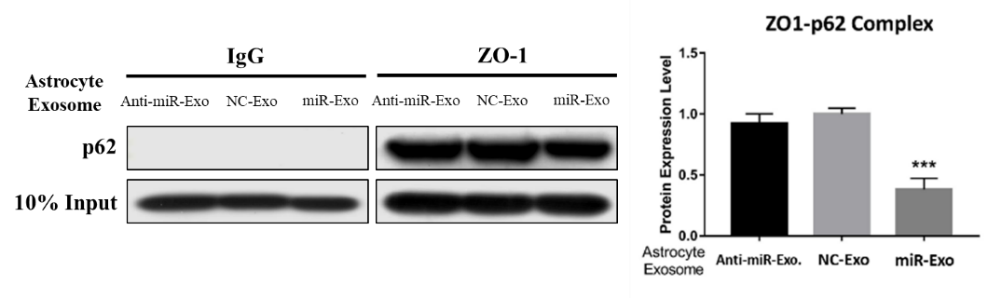
(2F)

**Supplemental Fig 2. miR-Exo increased TJ protein levels but had no effect on TJ mRNA.** (A) TJ proteins were measured at 72 h-post exosome treatment by western blot. Quantitative data from western blot are shown in the right using NC-Exo as the reference group. (B-C) TJ mRNA levels were not affected by the exosome treatment using NC-Exo as the reference group. (D) Autophagy-associated proteins p62, Beclin-1, and LC3-II / I were measured at 72 h-post exosome treatment by western blot. Quantitative data from western blot are shown in the right using NC-Exo as the reference group. (E-F) Co-immunoprecipitation (co-IP) assay was performed to detect the (E) p62/Claudin-5 and (F) p62/ZO-1 complex. Claudin-5 or ZO-1 were used as 10% input loading control. The co-IP results are shown in the left and the quantitative data in the right. Data A-F are presented as mean ± SEM from three independent experiments, ***p* < 0.01, and ****p* < 0.001.


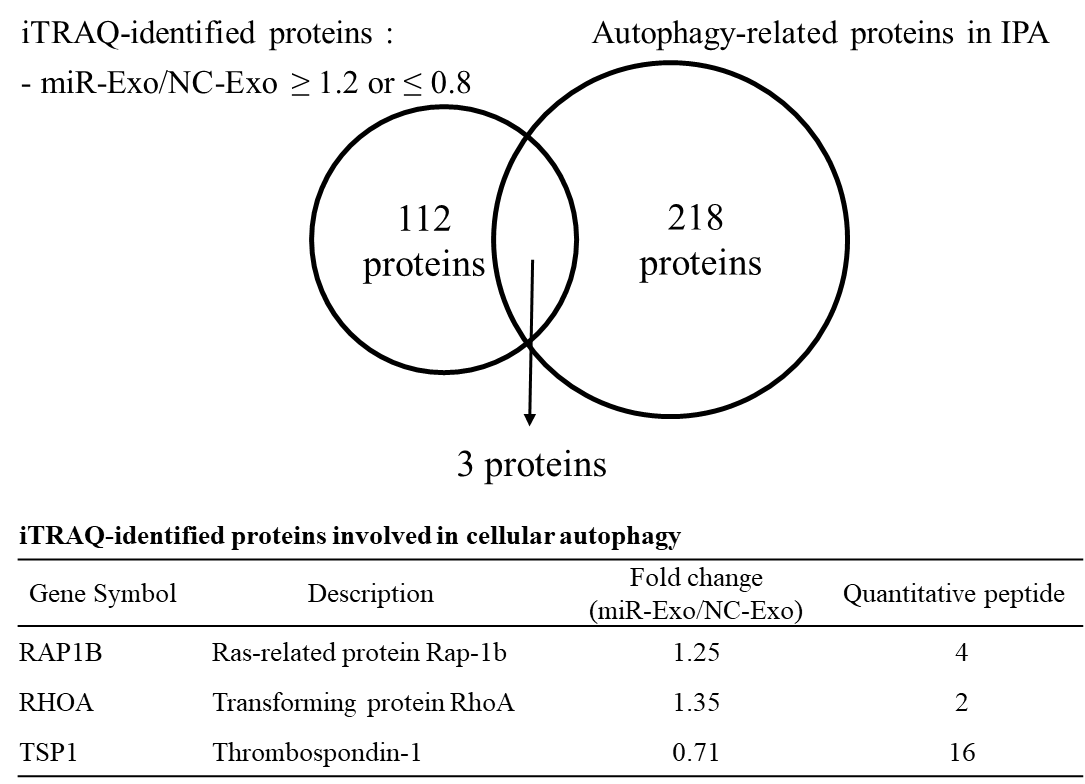
(3A)


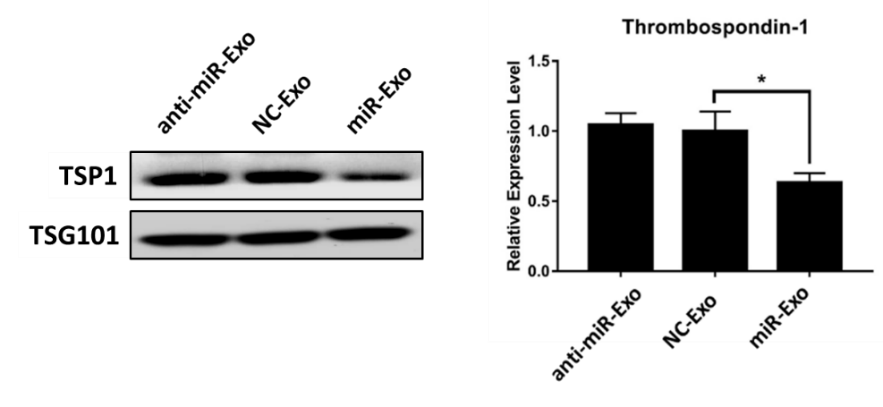
(3B)


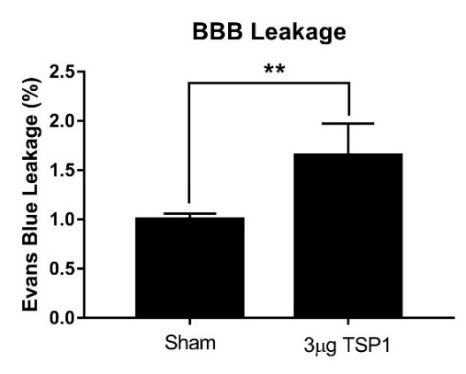
(3C) (3D)


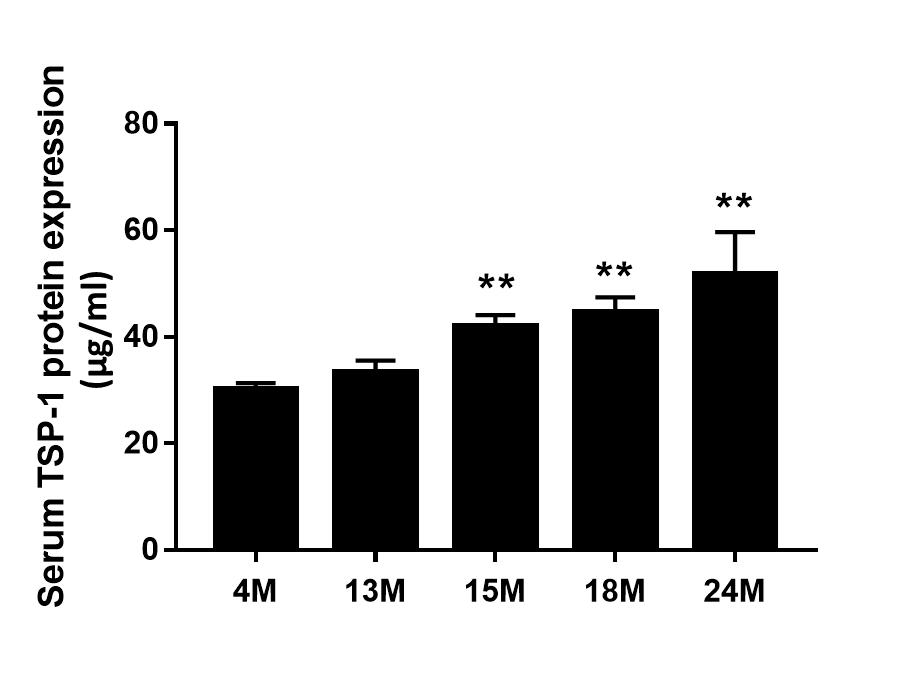


**
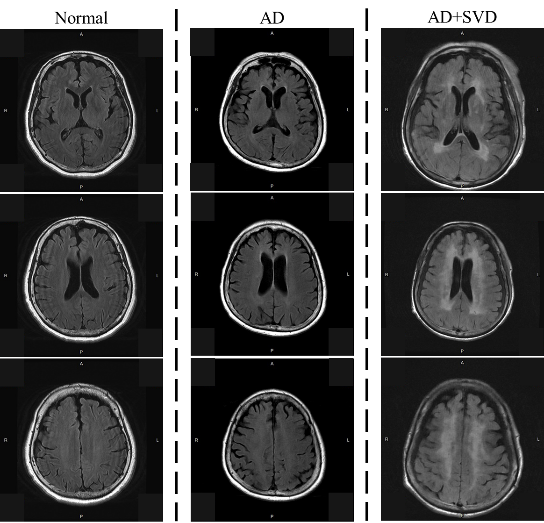
**(3E)

**Supplemental Fig 3. TSP1 is the key factor that induces BBB leakage.** (A) Screening for autophagy-related proteins in exosomes by the Ingenuity Pathway Analysis (IPA) database and iTRAQ analysis. The Venn diagram indicates intersected 3 autophagy-related proteins in miR-Exo with either the expression levels increased by 1.2-fold or decreased by 0.8-fold compared with NC-Exo. (B) Western blot demonstrates that TSP1 protein level was decreased in miR-Exo, which confirms the result of iTRAQ analysis. TSG101 was used as an exosomal loading control. The western data are shown in the left and the quantitative data are shown in the right. Data are presented as mean ± SEM from three independent experiments. (C) TSP1 was intracerebroventricular (ICV) injected to the mice and Evans Blue assay was performed on day 5. n=3 per group, ***p* < 0.01. (D) Serum TSP1 measured by ELISA was significantly increased in the aged mice. n=3 per group, ***p* < 0.01. (E) Representative MRI images of patients with and without small vessel disease (SVD). Normal, healthy control; AD, Alzheimer’s disease; SVD, small vessel disease. See serum TSP1 levels in figure 3G.

(4A)


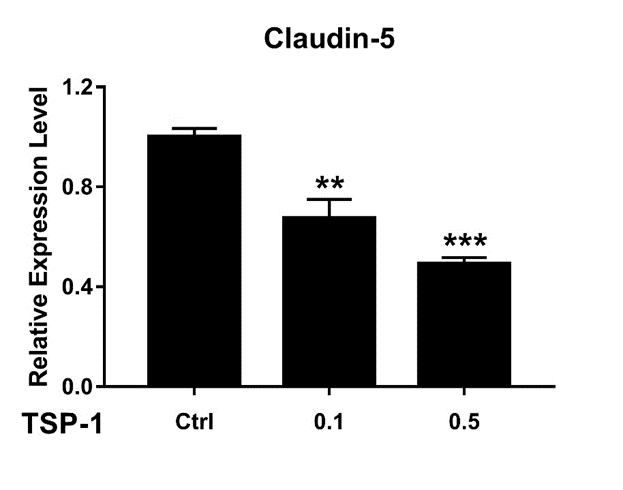

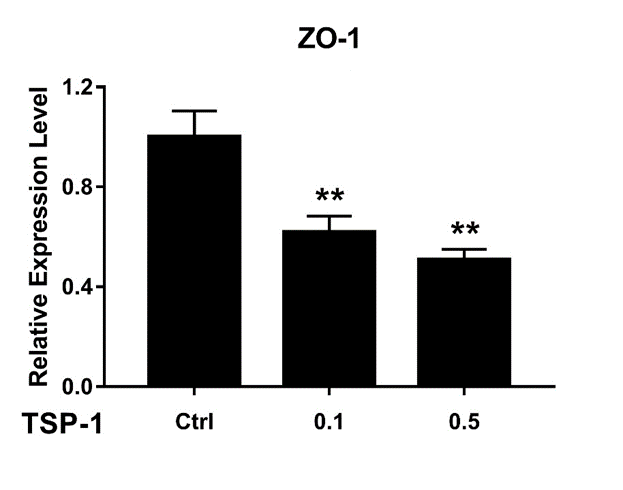

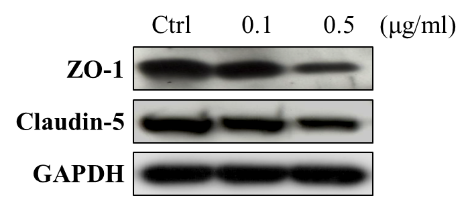


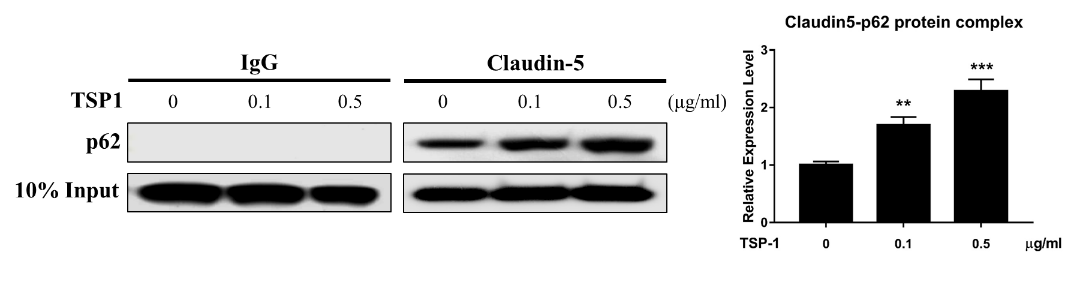
(4B)


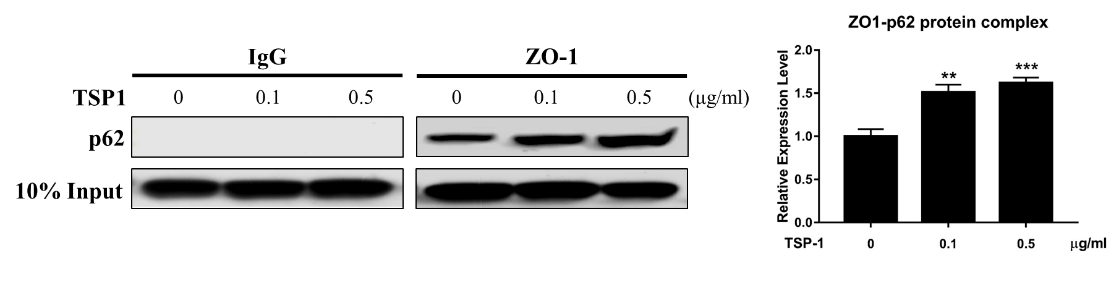
(4C)

(4D)


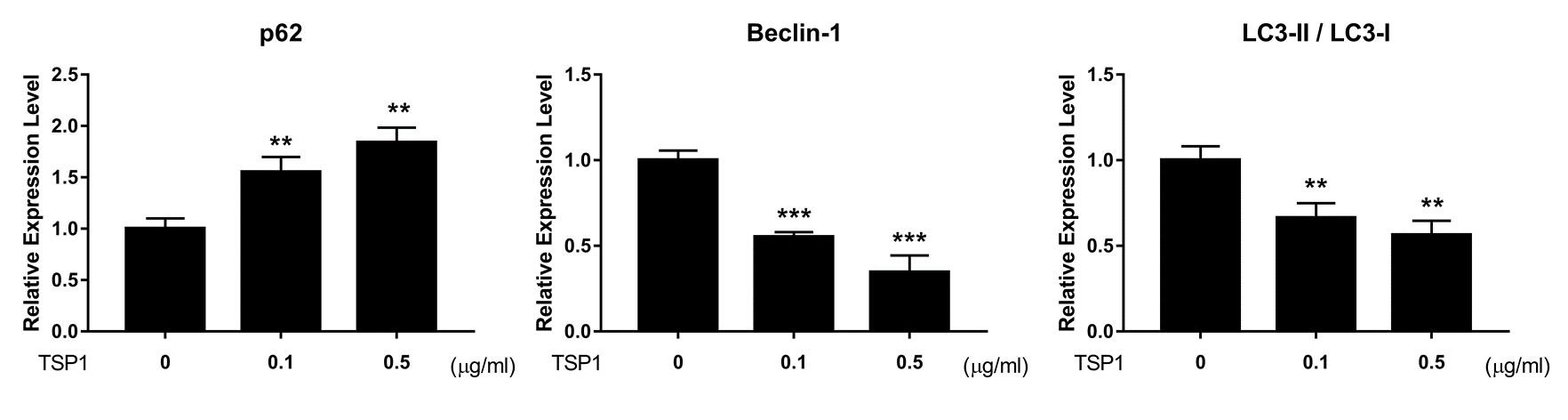

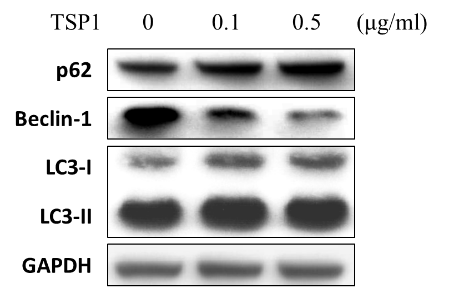


**Supplemental Fig 4. TSP1 suppresses TJ proteins expression by activating selective autophagy.** (A) TJ proteins at 72 h-post TSP1 treatment. Representative image of western blot is shown in the left and the quantitative data are shown in the right. (B-C) The co-immunoprecipitation (Co-IP) assay shows the formation of p62/TJ complex. ECs were treated with TSP1 and cell lysate was collected at 24 h for co-IP assay. Claudin-5 or ZO-1 were used as 10% input loading control. The co-IP data are shown in the left and the quantitative data are shown in the right. (D) Autophagy-associated proteins p62, Beclin-1, and LC3-II / I were measured at 72 h-post TSP1 treatment by western blot. Quantitative data from western blot are shown in the right. All quantitative data are presented as mean ± SEM from three independent experiments, ***p* < 0.01, and ****p* < 0.001.


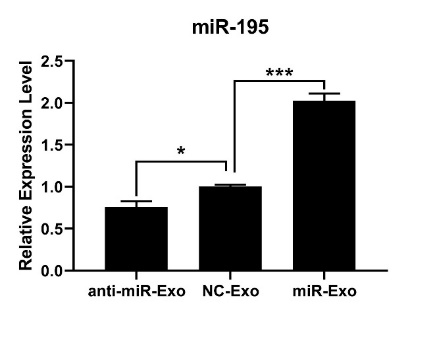
(5A)


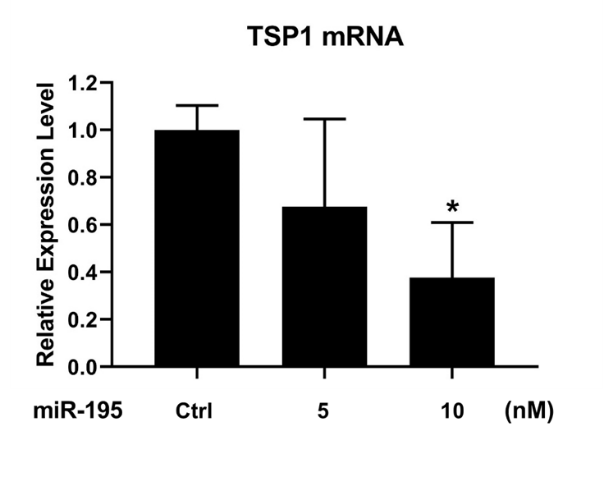
(5B) (5C)

(5D)


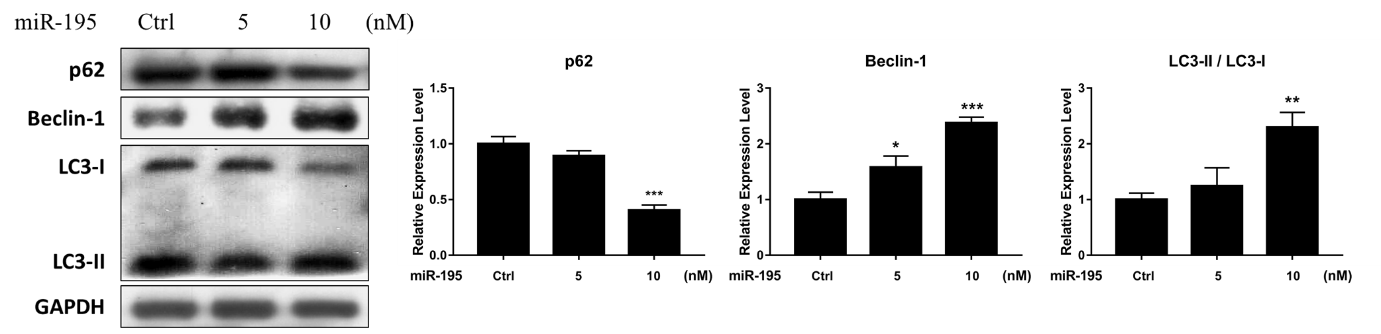


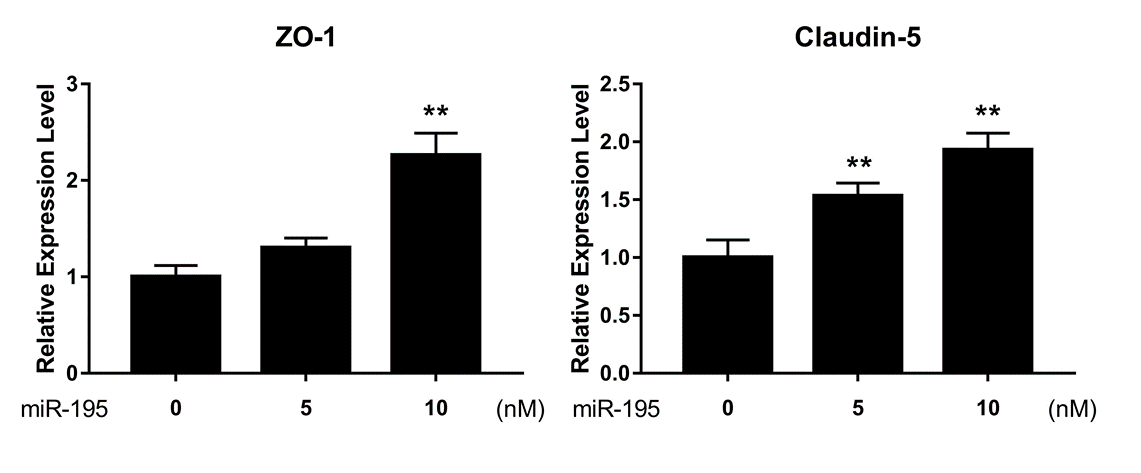

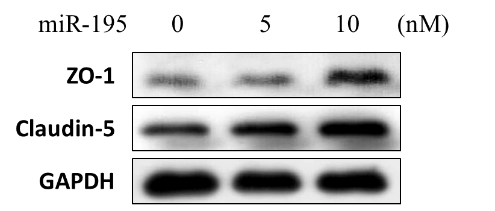
(5E)


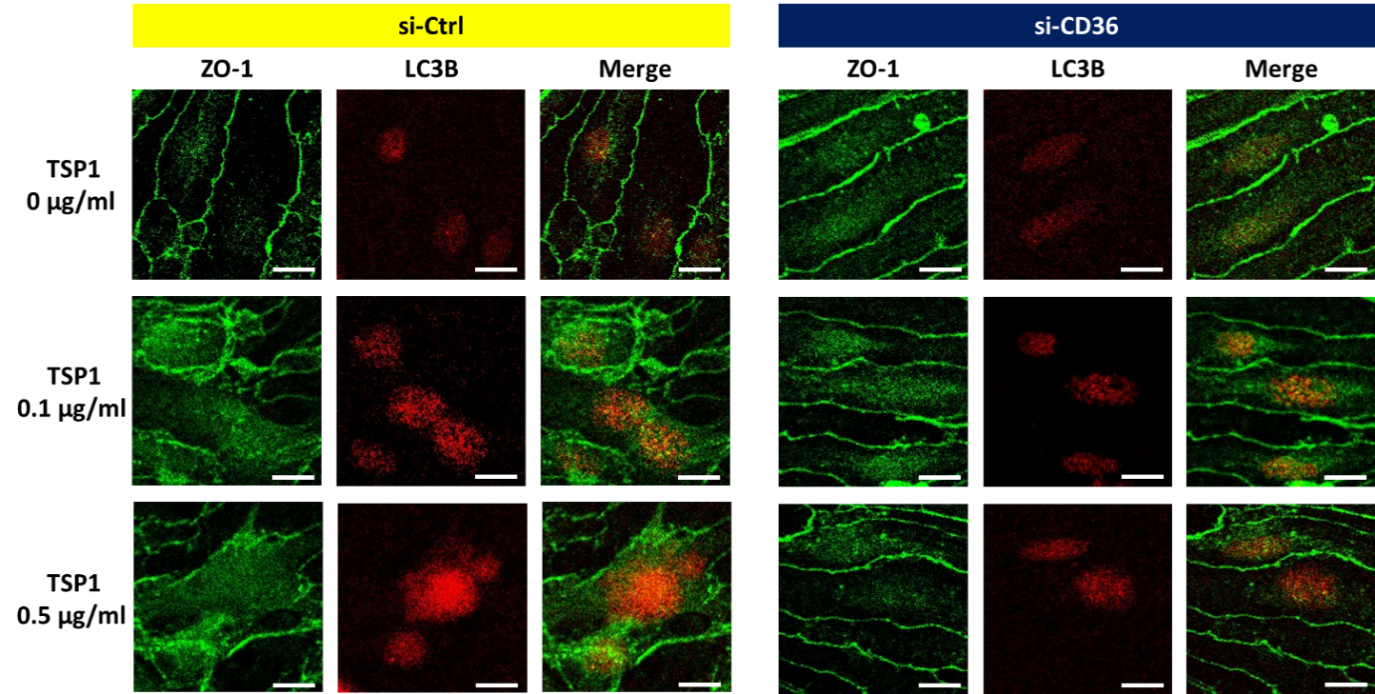
(5F)


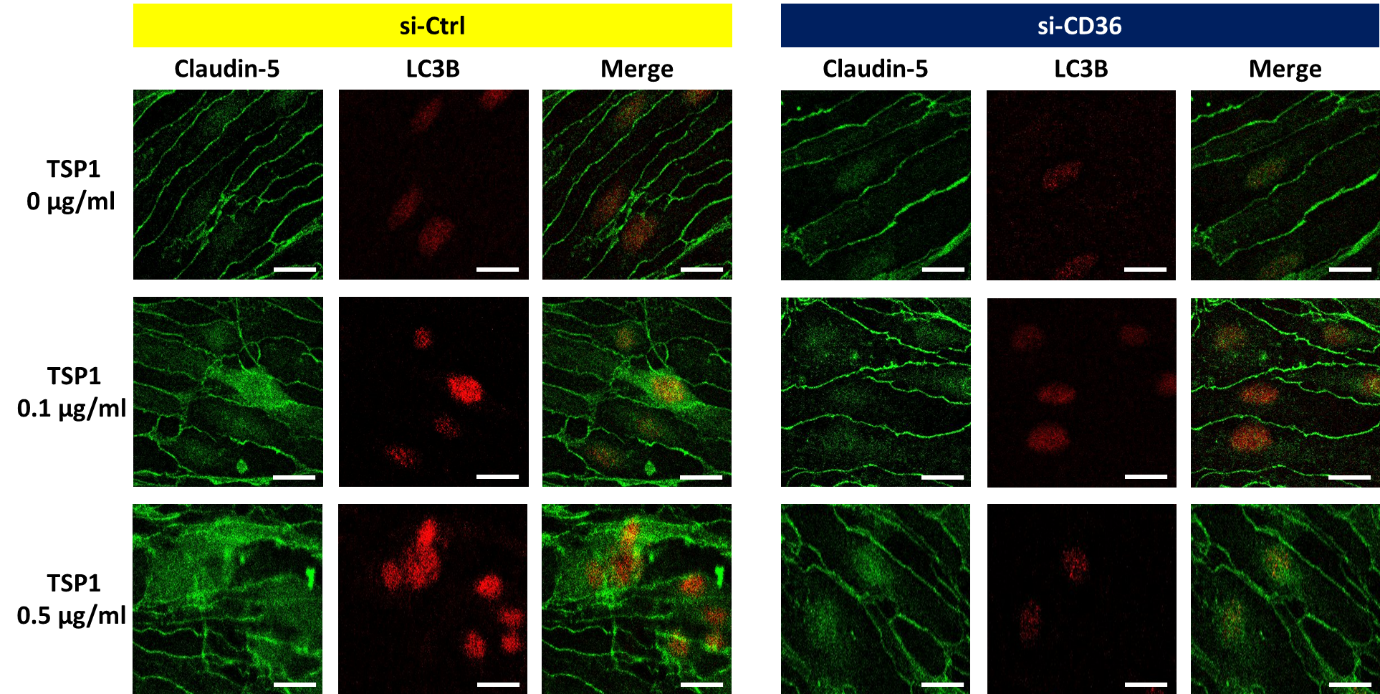


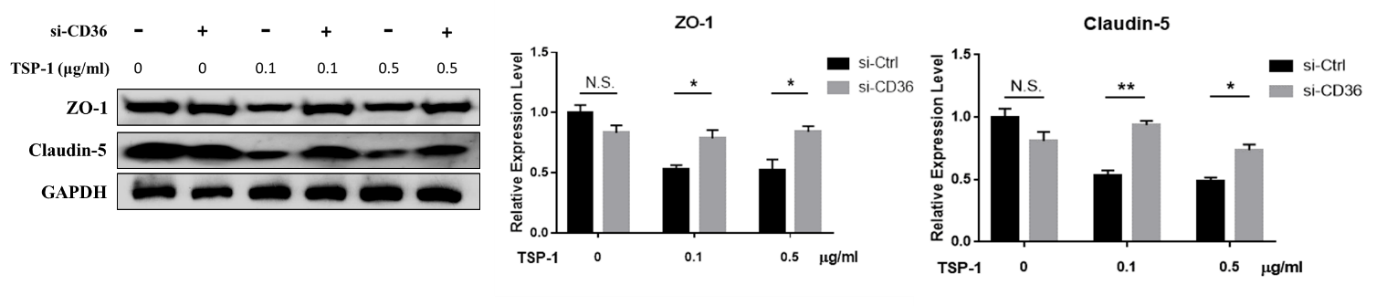
(5G)


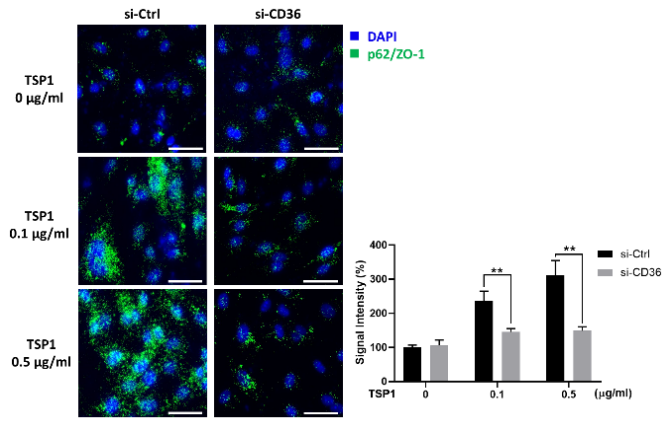

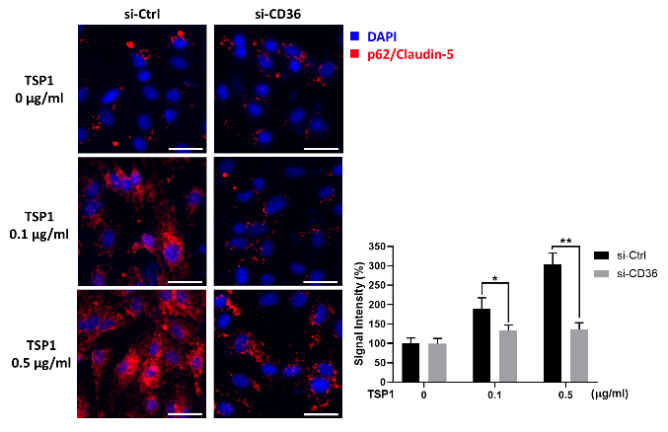
(5H)


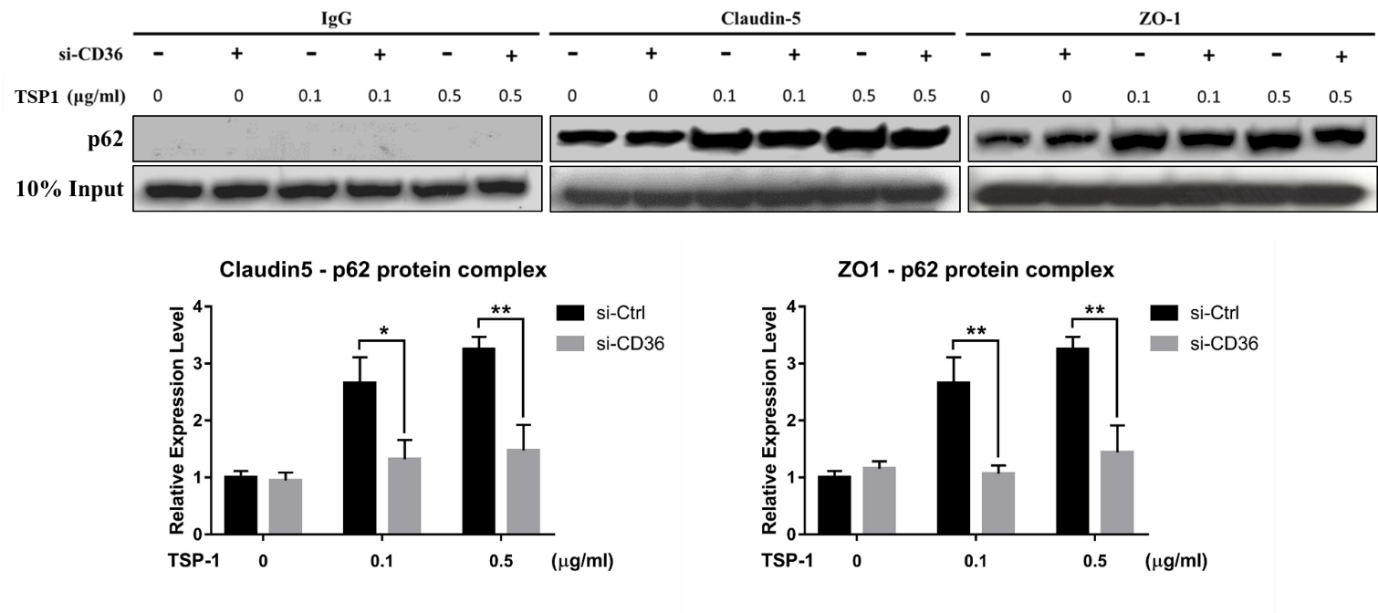
(5I)

**Supplemental Fig 5. miR-195-regulated TSP1 increased selective autophagy of TJ proteins via membrane receptor CD36.** (A) miR-195 levels in ECs at 6 h post-exosome treatment. (B) TSP1 mRNA levels in ECs at 24h after exosome treatment. (C) TSP1 mRNA levels in ECs at 24 h after miR-195 transfection. (D) Autophagy-associated proteins p62, Beclin-1, and LC3-II / I were measured at 72 h-post miR-195 transfection by western blot. Quantitative data from western blot are shown in the right. (E) TJ proteins were measured at 72 h-post miR-195 transfection by western blot. Quantitative data from western blot are shown in the right. (F) ECs were first transfected with si-CD36 or si-Ctrl for 48 h and subsequently treated with TSP1 for the next 24 h. Immunofluorescent images indicate that TSP1-induced TJ translocation was reversed in CD36-knockdown ECs. (G) Western blots of TJ proteins and their quantitative data from three independent experiments. (H-I) PLA and co-IP were performed to determine p62/TJ complex. ECs were treated with si-CD36 and TSP1 as mentioned in figure S5F. (I) The immunoblot of co-IP assay are shown in the upper panel and the quantitative data are shown in the lower panel right. Claudin-5 or ZO-1 were used as 10% input loading control. All data are presented as mean ± SEM from three independent experiments, **p*<0.05, ***p* < 0.01, and ****p* < 0.001 are calculated from the comparison with NC-Exo, control si-RNA or control miRNA treatments.
